# Cellular Uptake of Bevacizumab in Cervical and Breast Cancer Cells Revealed by Single-Molecule Spectroscopy

**DOI:** 10.1021/acs.jpclett.2c03590

**Published:** 2023-01-31

**Authors:** Aneta Karpinska, Gaweł Magiera, Karina Kwapiszewska, Robert Hołyst

**Affiliations:** † Department of Soft Condensed Matter, Institute of Physical Chemistry PAS, 01-224 Warsaw, Poland; ‡ Department of Medicine, Poznan University of Medical Sciences, 61-701 Poznan, Poland

## Abstract

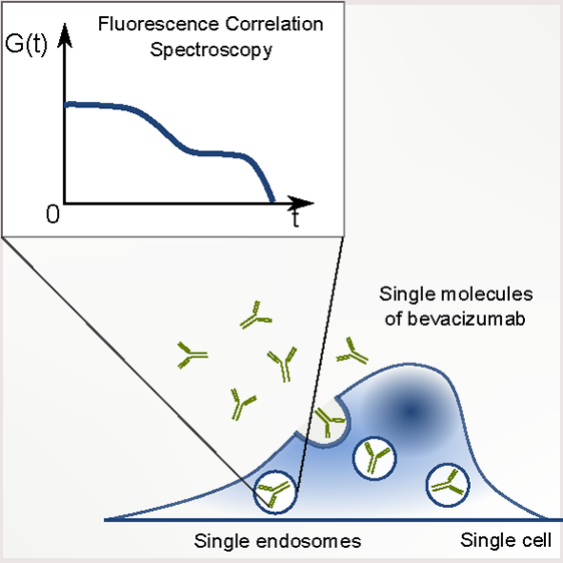

Bevacizumab is a biological drug that is now extensively studied in clinics against various types of cancer. Although bevacizumab’s action is preferably extracellular, there are reports suggesting its internalization into cancer cells, consequently decreasing its therapeutic potential. Here we are solving this issue by applying fluorescence correlation spectroscopy in living cells. We tracked single molecules of fluorescent bevacizumab in MDA-MB-231 and HeLa cells and proved that mobility measurements bring significant added value to standard imaging techniques. We confirmed the presence of the drug in intracellular vesicles. Additionally, we explicitly excluded the presence of a free cytosolic fraction of bevacizumab in both studied cell types. Extracellular and intracellular concentrations of the drug were measured, giving a partition coefficient on the order of 10^–5^, comparable with the spontaneous uptake of biologically inert nanoparticles. Our work presents how techniques and models developed for physics can answer biological questions.

In 2014, bevacizumab, an anticancer antibody, was first approved by the Food and Drug Administration to treat metastatic colorectal cancer. Since then, the drug has been approved for eight other cancer types. Today, there are several hundreds of active clinical trials of bevacizumab.^1^ Researchers are studying the use of this anticancer agent in more than 15 different cancers, including lung, breast, or cervical cancer. Considering the high therapeutic potential, bevacizumab seems to be a very promising drug in oncology. The simple scheme presenting the bevacizumab mechanism of action is presented in Figure 1A.

**Figure 1:**
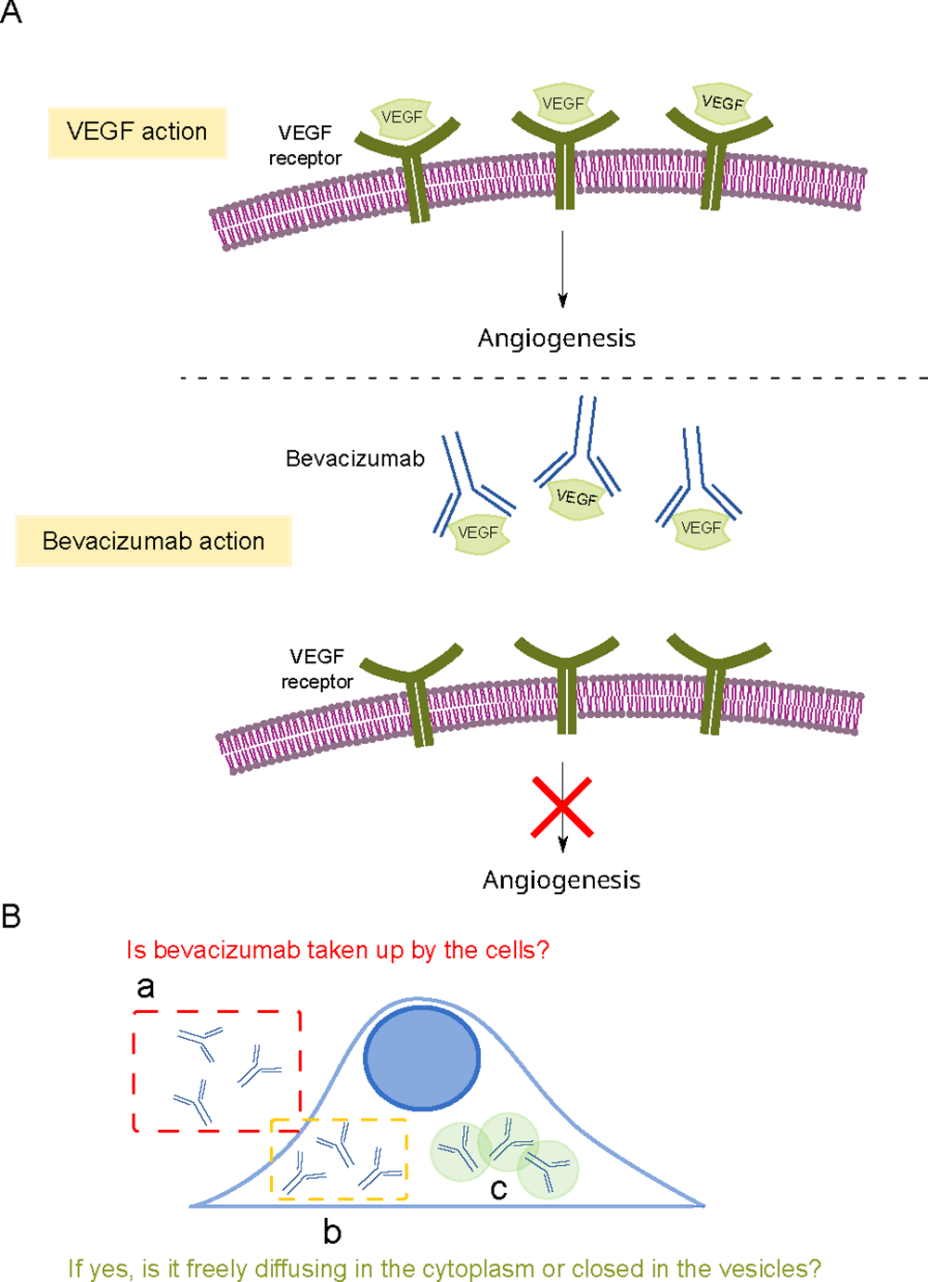
(A) Mechanism of bevacizumab action. The bevacizumab target is the VEGF. In the absence of the drug, the VEGF is bound to its specific receptors, initiating angiogenesis. When the bevacizumab is present, binding between VEGF and the receptor is inhibited as the bevacizumab binds to the VEGF. After the VEGF is attached to the drug, the VEGF cannot bind to the receptor. (B) Possible fractions of bevacizumab during the internalization studies. (a) The drug is not taken up by the cells and freely diffuses in the culture medium. (b) Bevacizumab is internalized into the cell and freely diffuses in the cytosol. (c) Bevacizumab is internalized into the cell but remains closed in the vesicles.

In targeted therapies, the drug must bind to a molecular target to develop a clinical response. Molecules like bevacizumab, acting outside of the cells, should not be internalized into the cell interior. Internalization of bevacizumab could even suggest nonspecific binding of the drug to the receptor or binding to a VEGF receptor previously associated with the drug. Thus, the chances of successful drug therapy can be impaired by its internalization properties (Figure 1B). Although bevacizumab is not supposed to get into cells, a few literature reports describe its penetration into the cell interior. For example, Deissler and others^2^ showed that bevacizumab internalizes retinal endothelial cells. More importantly, the authors indicated the drug binding to specific subcellular fractions: cytosol, membranes and/or organelles, and cytoskeleton.

Western blot and immunostaining results showed that a significant amount of the drug is bound to the cell cytoskeleton, depending on the fetal bovine serum (FBS) concentration in the culture medium.^2^ Similar results were published by Borchers and others in 2021.^3^ The main difference between these two literature reports was the incubation time with the bevacizumab. Borchers and co-authors tested weekly antibody penetration up to 12 weeks of incubation, distinguishing the additional subcellular fraction present in the cell nucleus. They simultaneously demonstrated that bevacizumab co-localizes with actin filaments.^3^ Another literature report on bevacizumab internalization concerned recurrent glioblastoma cells. The authors demonstrated that CD133+ cells *in vitro* internalize the tested drug by macropinocytosis after incubation for 5 min.^4^ All of the mentioned papers report the process of penetration of bevacizumab into cells. However, the techniques they used (static imaging or cellular content extraction) involved some biases that could have influenced the results. On the contrary, there are publications describing the novel technique for modifying anticancer drugs, such as bevacizumab, to enable crossing the cell membrane barrier.^5^ The formed, modified antibodies, called cytotransmabs, enter cells mainly by endocytosis, subsequently escaping from endosomes without being degraded. In both strategies, there is a need for the development of techniques providing data leaving no doubt on the subcellular localization of the drug.

Even when the drug is detected in the living cell interior, it is still unclear whether it crossed the cell membrane and was released to the cytosol or remained closed in vesicles. Here we propose a strategy for overcoming this limitation. We used two methods to investigate bevacizumab cellular uptake: fluorescence lifetime imaging (FLIM) and fluorescence correlation spectroscopy (FCS), each performed directly inside living cells nondisruptively. Our previous studies proved we can define whether the tested fluorescent cargo is freely diffusing in the cytoplasm, bound to its target, or undergoes oligomerization.^6−8^ We compare the diffusion coefficient of the cargo inside the cells with the diffusion coefficient predicted using the previously described length scale-dependent nanoviscosity models for cytoplasm^9,10^ and nucleus.^11^ If the obtained diffusion coefficient is smaller than the predicted one, the tested cargo interacts with the cells’ specific components. On the contrary, if the movement of the cargo is faster than we predicted, the possible phenomenon is the degradation of the probe. In addition, we can distinguish cargos freely diffusing in the cytosol from the cargos closed inside the vesicles. Suppose the molecule is present inside the vesicles. In that case, it is reflected in the shape of FCS curves and the physical model used to fit experimental data. Next to the commonly used method dedicated to analyzing cellular uptake, FCS provides data on a number of probe fractions in the cell environment, the concentration of each fraction, and the intracellular fraction type (Figure 1B). In addition, FCS, as a technique for collecting data within seconds, allows the real-time monitoring of cellular uptake. Conducting such studies before starting clinical trials will give us the guidance needed to design new drugs or drug delivery methods.

Using FLIM (section SI1 of the Supporting Information), we started the study of the internalization of bevacizumab into cervical cancer (HeLa) and triple-negative breast cancer (MDA-MB-231) cells. We incubated cells of both tested cell lines with 500 nM fluorescently labeled drug for 1, 24, and 48 h. After these periods of time, we performed imaging distinguishing the cell autofluorescence signal from the drug-derived signal using appropriate filtering, <2.4 and >2.6 ns, respectively.^7^

After incubation with the drug for 1 h, HeLa cells did not internalize bevacizumab. No drug-derived signal was observed inside cervical cancer cells. The dark areas seen in Figure 2 (left panel) correspond to the cells. The red signal around the cells represents the drug. We observed the difference after incubation for 24 h. After this time, vesicles emitting a signal from Atto 488, the dye used to label bevacizumab, were present in the cells. Moreover, elongated structures around the cell nucleus were also stained (Figure 2; for more images, see section SI2 of the Supporting Information). These vesicles were also localized within the cell nucleus. Finally, after incubation with bevacizumab for 48 h, HeLa cells changed their morphology from cells with an elongated shape (characteristic for adherent cells) to cells with a spherical shape. The morphological observations suggested HeLa cell death after 48 h with the drug present in the culture medium.

**Figure 2:**
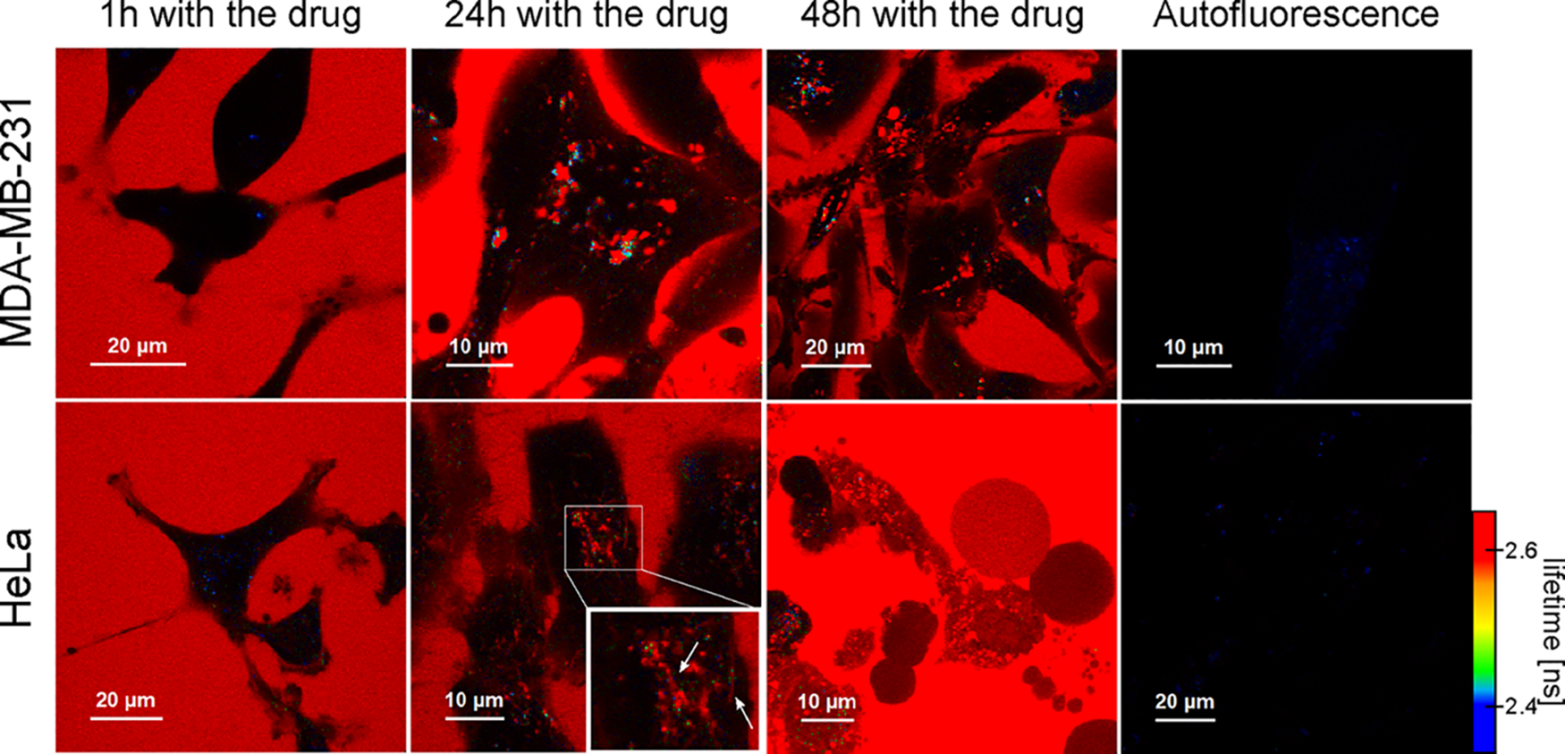
FLIM of HeLa and MDA-MB-231 cells incubated with bevacizumab for 1 h, 24 h, and 2 days. The red signal represents the tested drug, while the blue color corresponds to cell autofluorescence. In the case of HeLa cells after incubation with fluorescent bevacizumab for 24 h, the arrows indicate the elongated stained structures. Two independent experiments were conducted for each cell line within each incubation time.

Triple-negative breast cancer cells, MDA-MB-231, like HeLa cells, did not take up the drug after incubation with fluorescently labeled bevacizumab for 1 h. As in the case of the HeLa cell line, vesicles emitting a signal from Atto 488 attached to the tested drug were present inside the MDA-MB-231 cells after 24 h. However, at this stage, we noticed a difference between the two tested cell lines. In MDA-MB-231 cells, we did not see any stained, elongated structures that were observed in HeLa cells. Moreover, there was also a difference in the location and/or distribution of the vesicles. In MDA-MB-231 cells, the vesicles were more scattered within the whole cytoplasm. Furthermore, after 48 h, MDA-MB-231 cells remained viable (morphological assessment).

After collecting FLIM images, we quantitatively analyzed these data (Figure 3). We examined the fluorescence intensity coming from the blue channel with fluorescence lifetimes of <2.4 ns and the red channel representing fluorescence lifetimes of >2.6 ns. In this way, we demonstrated that the signal from the cytosol is significantly lower than the intensity emitted from the drug-filled vesicles (normalized to the extracellular average). It concerned both tested cell lines. Such quantitative analysis also proved the presence of elongated stained structures visible inside the interior of the HeLa cells after incubation with the tested drug for 24 h (fluorescence intensity record of cytosolic fraction seen in Figure 3F). In the case of MDA-MB-231 cells, we did not note any significant changes in the intensity of the areas of the vesicles and cytosol between 24 and 48 h incubation (Figure 3G). The same type of analysis was also performed for HeLa cells incubated with bevacizumab for 1 h. We proved that, after that time, there were not stained vesicles and the drug was not released into the cytosol (Figure 3D). Autofluorescence (blue channel) at certain points within the analyzed linear ROI significantly exceeded the fluorescence record for Atto 488-labeled bevacizumab (red channel). For more detailed FLIM analysis, see section SI3 of the Supporting Information.

**Figure 3:**
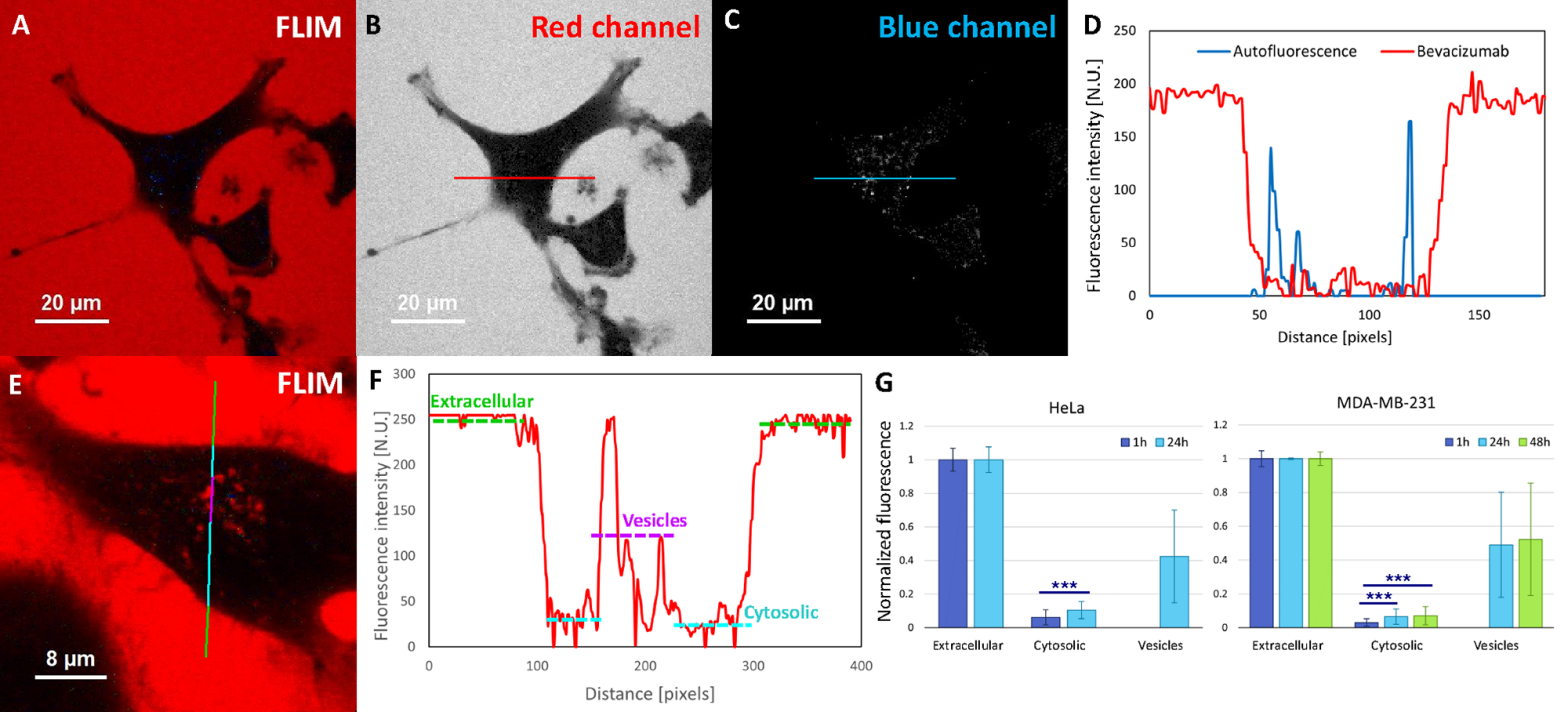
Quantitative analysis of FLIM images. (A) FLIM image of HeLa cells incubated with bevacizumab for 1 h, with colors representing fluorescence lifetimes: blue for autofluorescence (fl < 2.4 ns) and red for bevacizumab (fl > 2.6 ns). (B) Red channel extracted from image A, with the linear ROI marked. (C) Blue channel extracted from image A, with the linear ROI marked. (D) Fluorescence intensity profiles of the ROIs. (E) FLIM image of HeLa cells incubated with bevacizumab for 24 h, in which the linear ROI was marked with coloring corresponding to the labels in panel F. (F) Fluorescence intensity profile of the red channel of image E. Regions of extracellular, cytosolic, and vesicle areas are marked. (G) Averaged values of fluorescence intensity determined from FLIM images. Four images were captured for each cell line and incubation time. Averaged values came from three measurements taken from each image. The number of analyzed data points was >300. For the analysis, we used ImageJ version 1.53a. Three asterisks denote a statistically significant difference (*p* < 0.0005).

Our obtained FLIM results stay in opposition with the literature. In any tested cell lines, we did not observe cytosolic fractions of bevacizumab reported by Deissler or Borchers.^2,3^ In the case of cervical cancer and triple-negative breast cancer cells, the drug was neither present in the cytosol nor associated with membranes or the cytoskeleton. However, the differences between our results and the literature data may be directly due to the type of tested cells. Deissler and Borchers investigated retinal endothelial cells. Moreover, we analyzed fluorescent bevacizumab’s internalization into living cells without altered metabolism. In the literature, there are data concerning fixed by formaldehyde cells, and immunostaining is the method used to study cellular uptake. Therefore, researchers track the internalization process indirectly. They see the fluorescent signal coming from the labeled secondary antibody (drug–primary antibody–secondary antibody–dye). In FLIM, we analyze the drug–dye conjugate. Nevertheless, we assume that the dye is attached to the tested molecule in both cases. For this reason, we performed numerous control experiments to ensure we were analyzing the bevacizumab–Atto 488 complex. Using FCS, we excluded the presence of free dye in the sample and determined the size of the tested dye–drug conjugate, which is ∼10 times larger than the dye itself (for more details, see section SI4 of the Supporting Information). We performed such tests each time before starting the incubation of cells with the drug.

FCS measurements inside the living cells allow the identification of single fluorescent molecules based on their diffusion times. The absence of FCS autocorrelation curves indicates the lack of the analyzed molecule inside the cell or the absence of a mobile fraction moving in the confocal focus. We performed FCS measurements coupled with FLIM at the single-cell level, positioning the confocal focus in two spots within every single cell: (1) where no signal from the fluorescent drug was visible in the imaging (within the cytosol) and (2) where the stained vesicles were present (around the nucleus in the case of HeLa cells).

From the places within the cytosol where no signal from the drug could have been seen on the FLIM images, we did not obtain FCS autocorrelation curves. The obtained signal symmetrically oscillated around 0, confirming that free fluorescent bevacizumab was absent in the cytosol. In addition, the quantitative analysis of FLIM images proved that the fluorescence intensity within the cytosol was significantly lower than that of the extracellular and vesicles areas (Figure 3G). We found this to be true for both tested cell lines.

In comparison, we recorded diffusion of bevacizumab enclosed in the vesicle at places where stained vesicles and elongated structures (in the case of HeLa cervical cancer cells) were present. Indeed, the movement of the probe inside the vesicle is evidenced by the obtained FCS autocorrelation curves. The obtained FCS autocorrelation curves were fitted with eq S5. By fitting, from each FCS curve, we obtained information about the diffusion coefficient of the vesicle, the diffusion coefficient of the drug encapsulated in the vesicle, additional velocity forcing vesicle motion (active transport of the vesicle along microtubules), and the radius of the vesicle. We showed the table with the values of all of the parameters mentioned in section SI5 of the Supporting Information. An exemplary FCS curve obtained at the vesicles site is shown in Figure 4. The average velocity of vesicles’ transport for both tested cell lines was 1.56 μm/s. The obtained value corresponds to the velocity of motor proteins of the kinesin family (including kinesin-1),^12^ transporters for endosomes along the microtubules.

**Figure 4:**
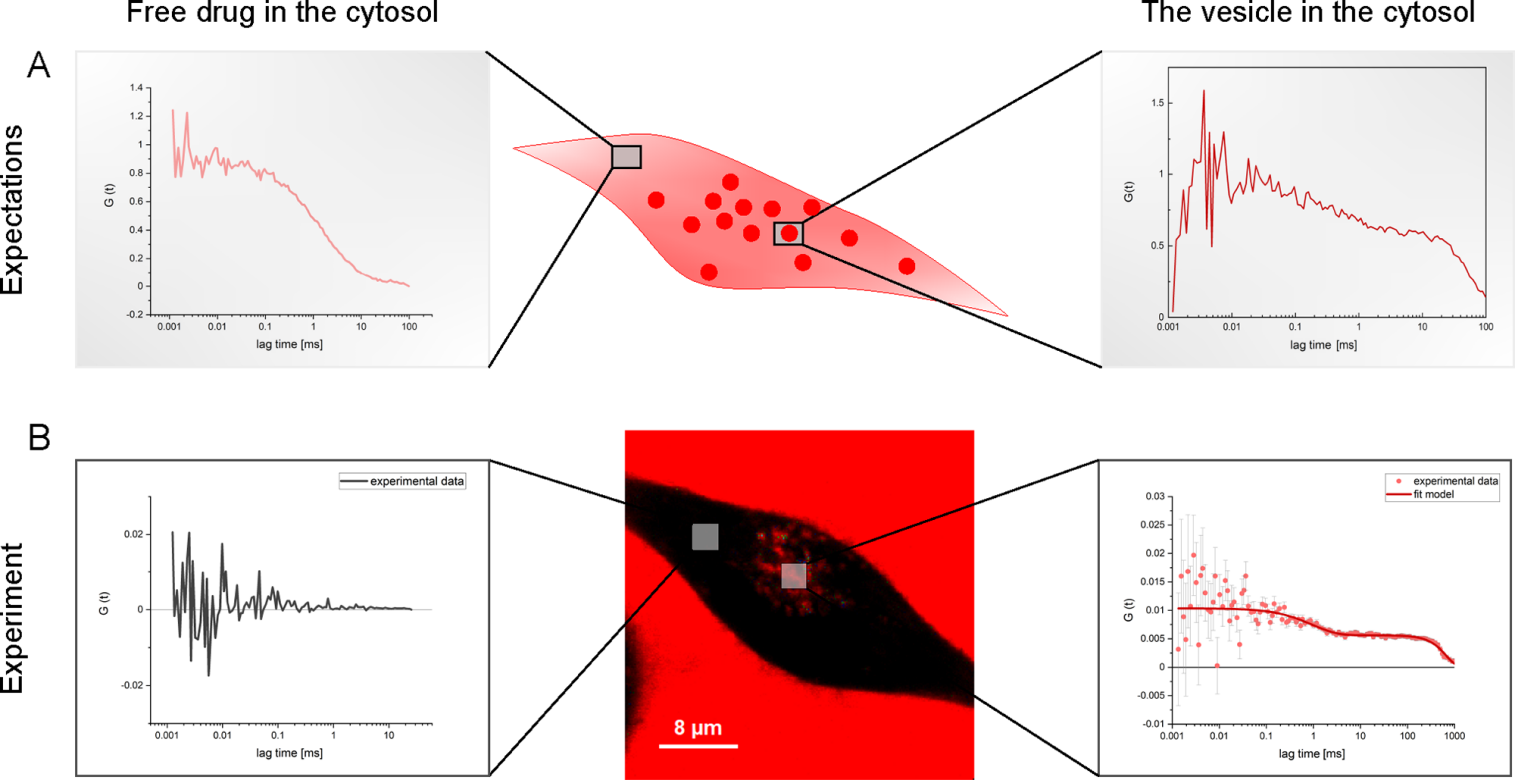
Comparison of expectations with actual experiments. (A) Scheme showing an example of FCS curves obtained inside a cell filled with the free drug and vesicles. The shape of the FCS curves varies depending on the position of the confocal focus (location of free drug vs location of vesicle). (B) Scheme presenting quantitative and qualitative results of the bevacizumab internalization study. The lack of the drug within the cytosol shown in FLIM was confirmed by the FCS measurements, the lack of an autocorrelation function. The presence of stained (drug-filled) vesicles was quantitatively proven by obtaining the FCS curves presenting the movement of the drug closed in the vesicle and the movement of the vesicle. The data were fitted using eq S5.

We confirmed the results obtained by FLIM/confocal imaging through FCS measurements. We excluded the presence of free, freely diffusing bevacizumab inside living cells. We also proved that single bright spots correspond to vesicles, endocytic vesicles or lysosomes, which are carried with active transport inside the cytoplasm.

The FCS measurements coupled with FLIM results clearly reveal differences in the intracellular fate of the bevacizumab entering different cancer cells. For both cell lines, the drug enters the cell by constitutive endocytosis. Undoubtedly, the drug is not transported by active uptake. If the drug were transported by endocytosis and/or pinocytosis, cells would be filled with many vesicles after a relatively short incubation time (sections SI6 and SI7 of the Supporting Information). The number of vesicles visible on FLIM/confocal images suggests that the drug enters the cell accidentally because endocytosis can be a process that occurs all of the time.^13^ The parameter that also excludes the active bevacizumab uptake is the time after which the vesicles are visible. In the case of active uptake, vesicles should be noticeable after incubation of the drug with the cells for only 1 h (Figure S5). In the case of bevacizumab, vesicles were not present inside both tested cell types until after incubation for 24 h.

The observed differences between the studied tumor types are likely associated with an extensive endomembrane system. The bevacizumab closed in the endocytic vesicle is digested in the lysosome, which is formed when the endocytic vesicle fuses with a Golgi vesicle containing digestive enzymes. The stained membrane structures around the nucleus in the case of HeLa cells probably correspond to membranes of the endoplasmic reticulum (ER). It is known that vesicular transport occurs between the endoplasmic reticulum and the Golgi apparatus.^14^ Bevacizumab may enter the ER through the endomembrane system (by retrograde transport),^15^ thus staining its membranes. It is also likely that the ER membranes are stained by the dye itself released after degradation of the antibody in lysosomes. Such a situation does not occur in MDA-MB-231 cells, in which the contents of endosomes and/or lysosomes do not enter the ER.

Using the possibility of determining concentrations with the FCS technique, we calculated the partition coefficient as the ratio of the bevacizumab concentration inside the cells to the drug extracellular concentration. In this way, we characterized the effectiveness of bevacizumab’s constitutive endocytosis. We determined the partition coefficient for both tested cell lines after incubation with the drug for 24 h and also 48 h in the case of MDA-MB-231. After such a time, we assumed that the exchange equilibrium (mechanism of uptake and removal) of the tested molecule between the cell and the medium had been reached. The obtained coefficients and the scheme of their calculation are summarized in Figure 5. The exact values of the coefficients are also included in Table S2. The concentration of bevacizumab closed inside intracellular vesicles was ∼10^5^ times smaller than the drug extracellular concentration. However, we hardly know whether, after such a relatively long time, the drug–dye complex is closed in the intracellular vesicles or the dye itself, as the antibody has been degraded. The partition coefficients were not significantly different for both tested cancer cells. In addition, in the case of MDA-MB-231 cells, the coefficients were very similar after incubation for 24 and 48 h. The constitutive endocytosis process seems thermodynamically dependent, as we did not note meaningful differences between cancer types and incubation times. It is worth mentioning that if the drug was transported in an active way and the contents of the vesicles were released into the cytosol, then the partition coefficient could reach a value close to or equal to 1. Indeed, one can assume that such a mechanism would be similar to the cargo delivery through osmotic shock published by Karpinska and others,^16^ where endocytosis is enhanced, and the cargo is released inside the cell.

**Figure 5:**
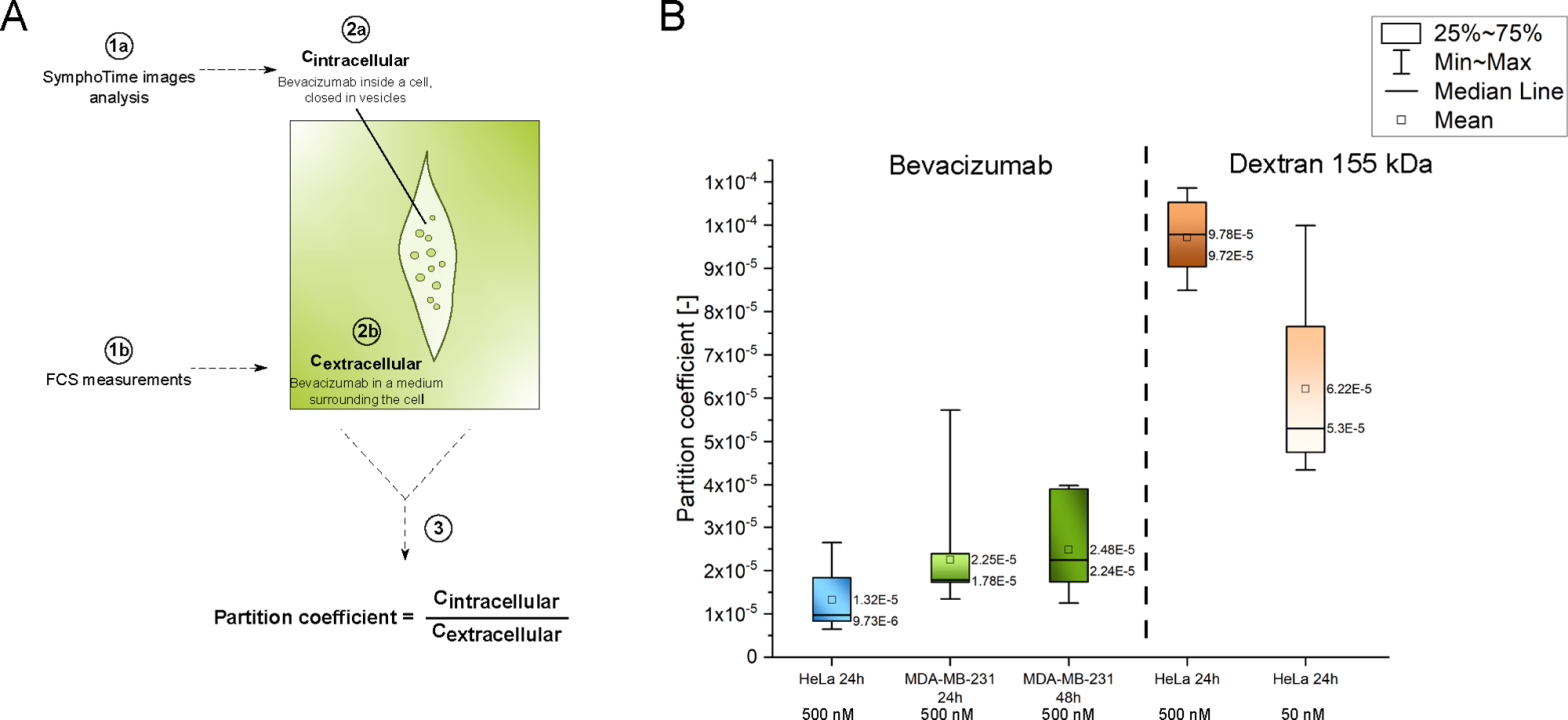
Partition coefficients as a parameter defining the effectiveness of bevacizumab internalization. (A) Scheme explaining the way of partition coefficient calculation. Parallel, intracellular, and extracellular drug concentrations were calculated (2a and 2b) using SymphoTime images analysis (1a) and FCS measurements (1b), respectively. Then, the partition coefficient was expressed as a ratio of the drug concentration inside the cells to the extracellular concentration (3). (B) Box plots of obtained partition coefficients for bevacizumab (HeLa after incubation for 24 h and MDA-MB-231 after 24 and 48 h) and TRITC-dextran 155 kDa (HeLa after incubation for 24 h with 500 nM and 50 nM) as a control. The results are averaged from eight independent repetitions. No significant differences between results from different cell lines were detected (*t* test; *p* > 0.05).

The calculated partition coefficient is approximately 100 000 times lower than that obtained for olaparib, the drug freely diffusing through the cell membrane.^6^ Such a difference is not unusual, as it is due to the mechanism of the transport of the drug into the cell. Olaparib, as a small molecule, freely penetrates by passive diffusion, accumulating inside the cells. In contrast, bevacizumab is not taken up by cells by diffusion or active endocytosis. The presence of the tested drug in cells is a consequence of constitutive endocytosis, occurring all of the time.

We compared the partition coefficient obtained for bevacizumab with a cell-neutral, non-cell-interacting polymer TRITC-dextran conjugate with a molecular weight of 155 kDa. We chose a conjugate with a hydrodynamic radius similar to that of bevacizumab that is equal to 8.6 nm.^17^ The only possible mechanism of transport of dextran into the cell should be constitutive endocytosis (occurring all of the time), as in the case of bevacizumab. We performed measurements for HeLa cells after incubation with 500 and 50 nM TRITC-dextran 155 kDa conjugate for 24 h. The obtained results compared with the partition coefficient results for bevacizumab are summarized in Figure 5B. We obtained a slightly higher partition coefficient for TRITC-dextran, which shows that bevacizumab, acting outside of the cells, is not taken up by the cells more readily than the compound neutral to them, the TRITC-dextran conjugate.

The partition coefficient is a constant parameter for a given compound and cell line, independent of the compound concentration in the culture medium.^6^ The differences in partition coefficients between 500 and 50 nM TRITC-dextran are probably due to equipment limitations. The concentrations were determined using the FCS method dedicated to the analysis of nanomolar concentrations. At concentrations of 500 nM, the obtained coefficients are subject to considerable error, from which the difference between the coefficient for 50 and 500 nM may arise. However, it should be emphasized that the order of magnitude of the partition coefficient for the process of constitutive endocytosis in the case of bevacizumab and TRITC-dextran 155 kDa is the same.

In summary, we have resolved the issue of bevacizumab internalization using the FCS method, which provides data about cargo fractions inside the cell, each fraction’s concentration, and the cargo transport mechanism. In this way, we showed that bevacizumab is not released from the vesicles to the cytosol. The eventual effect of photobleaching on FCS autocorrelation curves has been excluded (see section SI8 of the Supporting Information). We proved that the endosomes present in the cells do not originate from active uptake of the drug but result from the process of constitutive endocytosis occurring continuously. Moreover, we noted differences between cervical cancer and triple-negative breast cancer. In the case of HeLa cells, we observed elongated stained structures [confirmed in quantitative analysis of FLIM images (Figure 3F)] after incubation with the tested drug for 24 h. Such elongated structures were not seen in MDA-MB-231 cells. Most probably, these structures correspond to the ER membranes. There was also a difference in the location of the vesicles. In the case of cervical cancer cells (HeLa), the vesicles were centralized mainly around the cell nucleus. In comparison, in MDA-MB-231 cells, endosomes and/or lysosomes were distributed throughout the cytosol (Figure 2 and Figure S2). We determined the effectiveness of the bevacizumab internalization, calculating the partition coefficient as the ratio of the bevacizumab concentration closed in the intracellular vesicles to the drug’s extracellular concentration. We showed that this parameter is independent of cancer types or incubation time. We extended the study to check the cytotoxicity of bevacizumab, comparing the fluorescently labeled drug with unlabeled bevacizumab. We showed that fluorescent bevacizumab at concentrations of 250 and 500 nM killed cervical cancer (HeLa) cells that we did not observe for triple-negative breast cancer cells (MDA-MB-231). In contrast, the nonfluorescent drug exhibited no cytotoxic effect against any tested lines. It proves that drug labeling alters its pharmacological properties, and bevacizumab with an attached dye molecule appears to be a promising target for clinical trials in cervical cancer treatment. Our research shows that single-molecule FCS can provide valuable, conclusive data in the field of internalization of the drug into cells.

## Supporting Information Available

Materials and methods, confocal imaging of HeLa and MDA-MB-231 cells, detailed FLIM analysis, size of fluorescent bevacizumab, lack of interaction between bevacizumab and FBS components, quantitative data of FCS measurements performed inside HeLa and MDA-MB-231 cells, osmotic shock-enhanced pinocytosis, active transferrin uptake, exemplary time traces and corresponding autocorrelation curves, photobleaching analysis, possible impact of ECM proteases and nanoviscosity changes on the bevacizumab size, fluorescence intensity of bevacizumab and its complex with VEGF, brightness method as a method for monitoring the drug–target interactions, calculation of bevacizumab intracellular concentration, labeled bevacizumab that is toxic to HeLa cells, cell height in a two-dimensional culture, and FLIM images of HeLa cells incubated with 100 nM bevacizumab (PDF)
