# Supplementary material for: Cellular Uptake of Bevacizumab in Cervical and Breast Cancer Cells Revealed by Single-Molecule Spectroscopy
Source: J Phys Chem Lett. 2023 Jan 31;14(5):1272–8. doi: 10.1021/acs.jpclett.2c03590 (PMC9923738; doi:10.1021/acs.jpclett.2c03590)
Supplement: Supplementary file 1 — jz2c03590_si_001.pdf [file jz2c03590_si_001.pdf]

Supporting Information

to

Cellular Uptake of Bevacizumab in Cervical and Breast Cancer Cells Revealed by Single-molecule Spectroscopy

Aneta Karpińska[1], Gaweł Magiera[2], Karina Kwapiszewska[1], Robert Hołyst[1]

[1]Department of Soft Condensed Matter, Institute of Physical Chemistry PAS, 01-224, Warsaw, Poland

[2]Department of Medicine, Poznan University of Medical Sciences, 61-701, Poznan, Poland

SI1. Materials and methods

Reagents

Bevacizumab (Avastin®) used to study its internalization process was ordered from Selleck Chemicals LLC (www.selleckchem.com, Houston, USA), catalog number: A2006. According to the manufacturer's recommendation, the undiluted solution was stored at 4˚C in the dark. The experiments were performed within three months of purchase to avoid antibody degradation. The drug solution was kept under sterile conditions.

We investigated the interactions between bevacizumab and VEGF-A using Recombinant Human VEGFA protein (ab55566) purchased from Abcam (Cambridge, UK). The storage temperature was -20˚C. We avoided freeze/thaw cycles to keep the protein active.

Cell culture

HeLa and MDA-MB-231 cell lines came from the American Type Culture Collection (ATCC, Manassas, USA). Both types of cancer cells were cultured in the complete growth medium, supplemented with 10% *v/v* fetal bovine serum (FBS, Gibco), L-glutamine 1% *v/v* (Sigma-Aldrich), and 1% *v/v* solution of streptomycin [10 mg/ml] and penicillin [10 000 U/ml] (Sigma-Aldrich). Cultures were maintained in a 5% CO_2 humidified atmosphere and at 37˚C. HeLa cells were cultured in Dulbecco's Modified Eagle Medium (DMEM) with 1g/L of glucose (Institute of Immunology and Experimental Technology, Wrocław, Poland). The culture medium for MDA-MB-231 was RPMI-1640 with sodium bicarbonate and without L-glutamine (Sigma-Aldrich). Cells were maintained in a logarithmic growth phase. Passages were performed using 0.25% Trypsin-EDTA solution (Sigma-Aldrich) and phosphate-buffered saline (PBS, Sigma-Aldrich). Cell detachment was controlled under light microscopy. Cells were grown on an 8-chamber cover glass Cellvis (*California*, United States) slide for FCS measurements and FLIM. All experiments with cells were performed 24h or 48h after cell seeding.

Drug labeling

The antibody labeling procedure was performed Atto 488 NHS ester dye (Sigma-Aldrich). Samples were purified using Zeba™ Spin Desalting Columns, 7K MWCO, 2 mL (Thermo Fisher Scientific, Waltham, Massachusetts, USA), and MPW 260 centrifuge from MPW MED. INSTRUMENTS (Poland).

Before labeling, the antibody was passed through a column to remove possible impurities. Then, an appropriate volume of Atto 488 NHS ester dye solution in DMSO was added to the antibody solution. The molar ratio between dye and the antibody was 4:1. After 1h incubation at room temperature, 0.5 M TRIS-HCl buffer pH 6.8 was added to the bevacizumab-dye solution in a 4-fold excess of the used dye volume. In the next step, we proceeded to purify the antibody from the free dye. The whole procedure of column antibody purification was performed according to the protocol of the column manufacturer. The filtrate contained purified labeled bevacizumab. Confirmation of the absence of dye in the bevacizumab sample after passing through the columns several times was performed using the FCS technique.

MTT assay

Cells were detached from the growth surface according to the standard cell passage procedure. The obtained cell suspension was diluted to approximately number – 5 000 cells/well (both for MDA-MB-231 and HeLa). The cell number was controlled with Countess II Cell Counter (Thermo Fisher Scientific). In the next step, cells were seeded into a 96-well plate (Greiner Bio-One). Then, we incubated cells for 24h at 37˚C to enable cell attachment. After that time, we removed the culture medium (specific for

the cell line). We added the non-fluorescent bevacizumab at the concentrations 2 – 1 000 nmol/L to the fresh cell medium. Five repeats were performed for each tested concentration. We also performed controls: blank – only medium without cells, positive control - cells not treated with bevacizumab and negative control - dead cells, toxicant - 1% Triton X-100 (Sigma-Aldrich). The number of repeats for the control was also five.

The prepared plate was incubated at 37°C for 48 hours. Next, we replaced the medium with a fresh culture medium including 1 mmol/L 3-(4,5-dimethylthiazol-2-yl)-2,5-diphenyltetrazolium bromide (MTT reagent, Thermo Fischer Scientific). We additionally added 1% Triton X-100 to the negative control. Cells were incubated for 4 hours at 37 °C. Then, we replaced the solutions with DMSO and incubated the plate for 10 minutes. The absorbance in each well was measured at 540 nm using a Synergy HTX multi-mode reader (BioTek).

Overall, we performed two independent MTT assays for both tested cell lines.

The percentage of viable cells at each drug concentration was calculated using Equation S1.

$$Viability\ [\%] = \frac{measured\ value - average_{negative\ control}}{average_{positive\ control} - average_{negative\ control}}$$

(Equation S1)

The obtained values from five replicates were then averaged.

AlamarBlue® assay

We used the AlamarBlue® dye according to the manufacturer's protocol (*General Method for Measuring Cytotoxicity or Proliferation Using alamarBlue® by Fluorescence, Bio-Rad*). We performed five controls per each assay: a) medium alone with the dye (blank), b) cells with alamarBlue® untreated with bevacizumab (positive control), c) dead cells with alamarBlue®, toxicant - 1% Triton-X 100 (negative control), d) a pure medium without dye and e) cells untreated with the tested drug without alamarBlue® (autofluorescence control). After 48 hours of cell incubation at 37˚C, we added 10% of the dye to the phenol red-free culture medium (to eliminate the background fluorescence of phenol red). Then, we incubated the plate at 37 °C for 2-4 hours (fluorescence intensity controlled hourly). After that time, the fluorescence in each well was measured at 590 nm (590/20 filter) for excitation at 560 nm (560/20 filter).

We determined the unlabeled drug cytotoxicity at concentrations 2 - 1 000 nM (double dilutions). Each concentration included five replicates. In contrast, labeled bevacizumab was tested at concentrations 1 - 500 nM (double dilutions). The number of replicates for each concentration was three.

Cell viability for each tested bevacizumab concentration was calculated using Equation S1.

We made a HeLa and MDA-MB-231 cell viability plot for the fluorescent drug using a logarithmic scale on the x-axis. In the case of IC_{50} concentration (HeLa cells), we fitted experimental data with a logistic dose-response function including four parameters (Origin2020b software).

FCS setup

We performed FCS measurements using a Nikon Eclipse TE2000U confocal microscope coupled with Pico Harp 300 FCS equipment (PicoQuant, Germany). Measurements were conducted using a 60x

objective (N.A. 1.2) with water immersion. Fluorescence emitted by Atto 488 dye was excited using a 481 nm pulsed diode laser. Single-Photon Avalanche Diodes (MPD and PerkinElmer) collected the fluorescence photons. In the optical path before the detector, a 488 long-pass filter (Chroma, USA) was positioned.

Before each FCS measurement, we characterized the confocal volume, performing the calibration. For all measurements in a buffer, we used a nanomolar solution of Rhodamine 110 in PBS solution. In the case of FCS measurements inside living cells, we provided a similar refractive index of calibration solution as a studied system (cell cytoplasm) using Rhodamine 110 in 2.5% w/w glucose in PBS.[1]

We maintained the set temperature (36 ± 0.5 ˚C) using a climate chamber (Okolab, Italy). We used the QiuckFit 3.0 software (DKFZ, Germany) to fit the experimental data (FCS curves) with the appropriate mathematical model.

FCS measurements in a buffer

Measurements in a buffer were understood as measurements outside the cell. Thus, these were both extracellular measurements performed in the culture medium surrounding the tested cells and control measurements in PBS solution. In all experiments, the laser power was 10 µW.

All collected data during experiments in a buffer were fitted using a one-component normal diffusion model, presented in Equation S2.

$$G\left(\tau\right) = \frac{1}{N}\left(1 + \frac{\theta_{trip}}{1 - \theta_{trip}} \exp\left(-\frac{\tau}{\tau_{trip}}\right)\right) \cdot \frac{1}{1 + \left(\frac{\tau}{\tau_D}\right)} \frac{1}{\sqrt{1 + \left(\frac{\tau}{\tau_D}\right)\frac{1}{\kappa^2}}}$$

(Equation S2)

where N is the overall particle number in confocal volume, θ_{trip} stands for a fraction of particles in a triplet state, τ_D is the diffusion time of the diffusing component, κ is the Gaussian aspect ratio used to approximate the focus. The κ parameter was determined during calibration and had one exact value, always within 5.5-6.5.

The fitted parameter was the diffusion time of the component. Based on the value of τ_D, the diffusion coefficient given by Equation S3 was determined.

$$D = \frac{{\omega_{xy}}^2}{4\tau_D}$$

(Equation S3)

where ω_{xy} is the radius of the measurement volume defined during calibration, usually in the range of 180-230 nm.

We calculated the hydrodynamic radius of the drug and the drug – VEGF complex using the Stokes-Sutherland-Einstein equation (Equation S4). In a sample containing both the antibody and the VEGF-A factor, the stoichiometric ratio was 1:2, respectively.

$$R_h = \frac{k \cdot T}{6 \cdot \pi \cdot \eta \cdot D}$$

(Equation S4)

where k is Boltzmann's constant, T is the absolute temperature, η corresponds to the solvent viscosity equal to 0.00075 [Pa·s] for PBS and 0.00071 [Pa·s] for water at 36˚C.

FCS measurements inside living cells

In the first step of cellular FCS measurements, the focal volume was positioned inside a particular part of the cell (approximately 2 µm above the glass) using the microscope imaging mode. The alignment accuracy was up to 0.5 µm. The precise positions of the confocal focus in the cell are shown in Figure S1. After the detective position (where vesicles were visible or where there was no signal from the drug in the cytosol, Figure S1) was determined, we turned on the FCS mode. SymphoTime software (PicoQuant, Germany) collected the fluorescence fluctuation record of the probe diffusing through the confocal volume. As a result of mathematical processing, the fluctuation record was presented in the form of an autocorrelation curve. Finally, we fitted an appropriate function of a physical model to the experimental data.

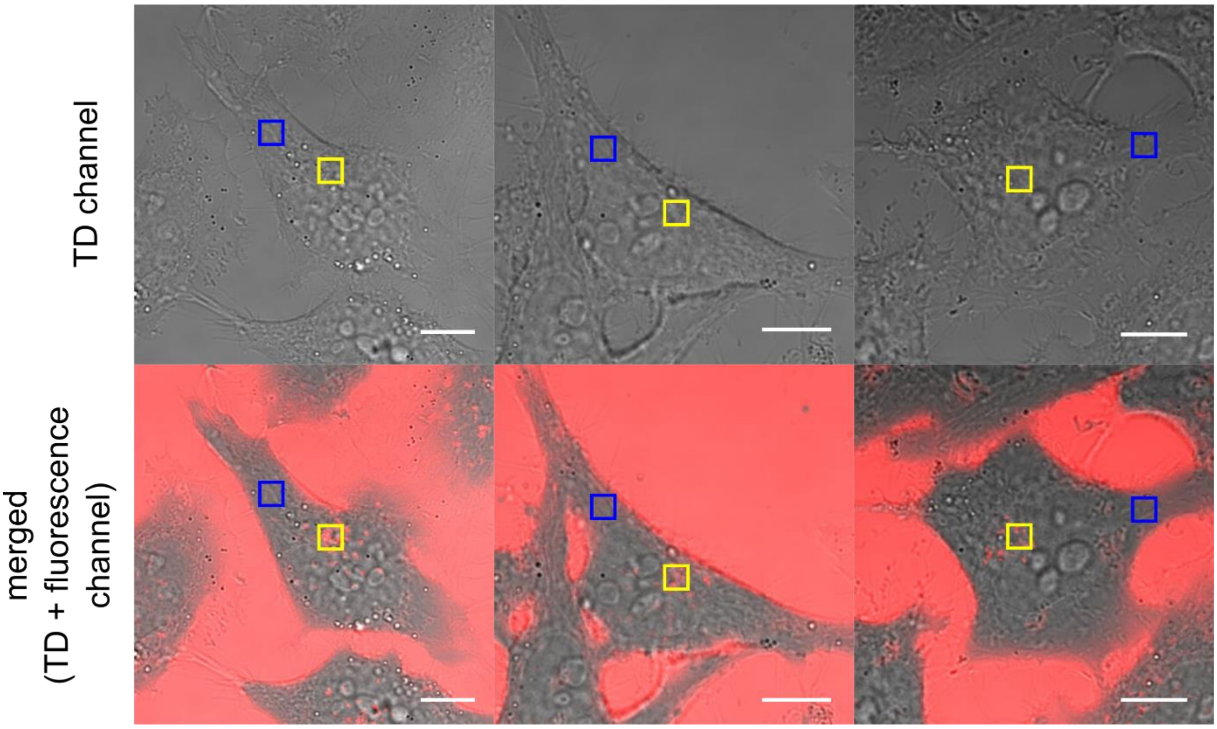

Figure S1. Confocal images of HeLa cells incubated 24h with 500 nM bevacizumab demonstrating the position of the confocal focus. A) Exemplary transmission images. B) Merged images (TD + fluorescence channel). The red signal is emitted by the tested drug. The blue square indicates the position of the confocal focus in the cytosol, yellow - at the site of vesicles. Scale bar = 10 µm.

We carried out measurements inside cells with spindle-shaped morphology. Ten cells were measured each time, taking three individual measurements per cell. The data acquisition time for single measurements was 20 s. In all experiments, the laser power was 10 µW as in a buffer.

FCS autocorrelation curves from the cell interior were fitted with the walking confined diffusion model described by Ochab-Maciolek and others,[2] to which vesicles' transport term was added. The fit model is presented in Equation S5.

$$G\,(t) = G_{inf} + \frac{1}{N}\left(1 + \frac{2}{3}\frac{\left[6D_M t + \frac{6}{5}a^2\left(1 - e^{-5D_m t/a^2}\right)\right]}{{\omega_{xy}}^2}\right)^{-1} x \left(1 + \frac{2}{3}\frac{\left[6D_M t + \frac{6}{5}a^2\left(1 - e^{-5D_m t/a^2}\right)\right]}{\kappa^2 {\omega_{xy}}^2}\right)^{-\frac{1}{2}} \cdot e^{-\left(\frac{tv}{\omega_{xy}}\right)^2}$$

(Equation S5)

Where N stands for the total particle number in the confocal volume, D_M corresponds to the diffusion coefficient of a vesicle, a – the vesicle radius, D_m is the diffusion coefficient of a probe enclosed in a vesicle, ω_{xy} stands for the radius of the measurement volume defined during calibration, and v is the velocity of vesicle motion. The κ parameter was determined during calibration and had one exact value, always within 5.5-6.5.

Fluorescence Lifetime Imaging (FLIM)

We performed FLIM using a FLIM mode of SymphoTime software. The time that the laser spends on every single pixel (pixel dwell) was equal to 60 µs. In all experiments, we took 10 scans. We applied filtering after fluorescence lifetimes because the used 488 nm laser also induces cell autofluorescence (mainly $FADH_2$ autofluorescence). Thus, a fluorescence lifetime of fewer than 2.4 ns was treated as autofluorescence. Pixels with an autofluorescence lifetime below 2.4 ns were colored blue, while pixels with a fluorescence lifetime above 2.6 ns were colored red. Using FLIM mode, we checked that the fluorescence lifetime for Atto 488 is above 3 ns. This value is consistent with the information from the manufacturer of the dye. Thus, the red signal came from the tested antibody (Figure 2).

Quantitative FLIM image analysis was performed using ImageJ software. First, the red channel was extracted from the full image and saved as a grayscale image. Next linear ROIs were determined, and grayscale value profiles were acquired. Values of the grayscale represented fluorescence intensity and were averaged over one time-points depending on the areas: extracellular, cytosolic, and vesicles. Data were normalized to the extracellular average to compare values for different time points.

Partition coefficient calculation

The partition coefficient[3] was calculated as the ratio of the bevacizumab concentration inside the tested cells to the bevacizumab extracellular concentration (in the culture medium surrounding the cells). FCS measurements determined the extracellular concentration of the drug. The intracellular concentration was calculated using time-dependent photon count data acquired with SymphoTime software, imaging module. Symphotime images were used to determine the number of photons per second per cell. Using bevacizumab molecular brightness (number of photons per second per molecule) obtained from the reference FCS measurement, we calculated the number of molecules inside the cells. Then, taking into account the cell volume (set as $2 \cdot 10^3$ µm^3), we calculated the intracellular concentration of bevacizumab closed inside the vesicles. A scheme explaining the way of intracellular concentration calculation is presented in Figure S9. The higher the partition coefficient, the better the endocytosis process's effectiveness.

Analogously, we performed measurements for TRITC-dextran 155 kDa (Sigma-Aldrich, product number T1287), well characterized in our previous study.[1]

Statistical analysis

Statistical analysis was performed using the Analysis ToolPak add-in to Microsoft Excel Software. The datasets were compared using a two-sample t-test assuming equal variances, α=0.05.

SI2. Confocal imaging of HeLa and MDA-MB-231 cells

We found differences in the bevacizumab internalization between MDA-MB-231 and HeLa cells. In the case of the HeLa cell line, after 24h, vesicles containing the drug or the dye used to label the bevacizumab were located around the nucleus. In addition, the fiber structures near or above the nucleus were stained. For comparison, after 24h, MDA-MB-231 cells, similarly to HeLa, contained the stained vesicles, but the vesicles were distributed around the whole cytoplasm. In Figure S2, we showed the exemplary confocal images of both tested cell lines to visualize the mentioned differences better.

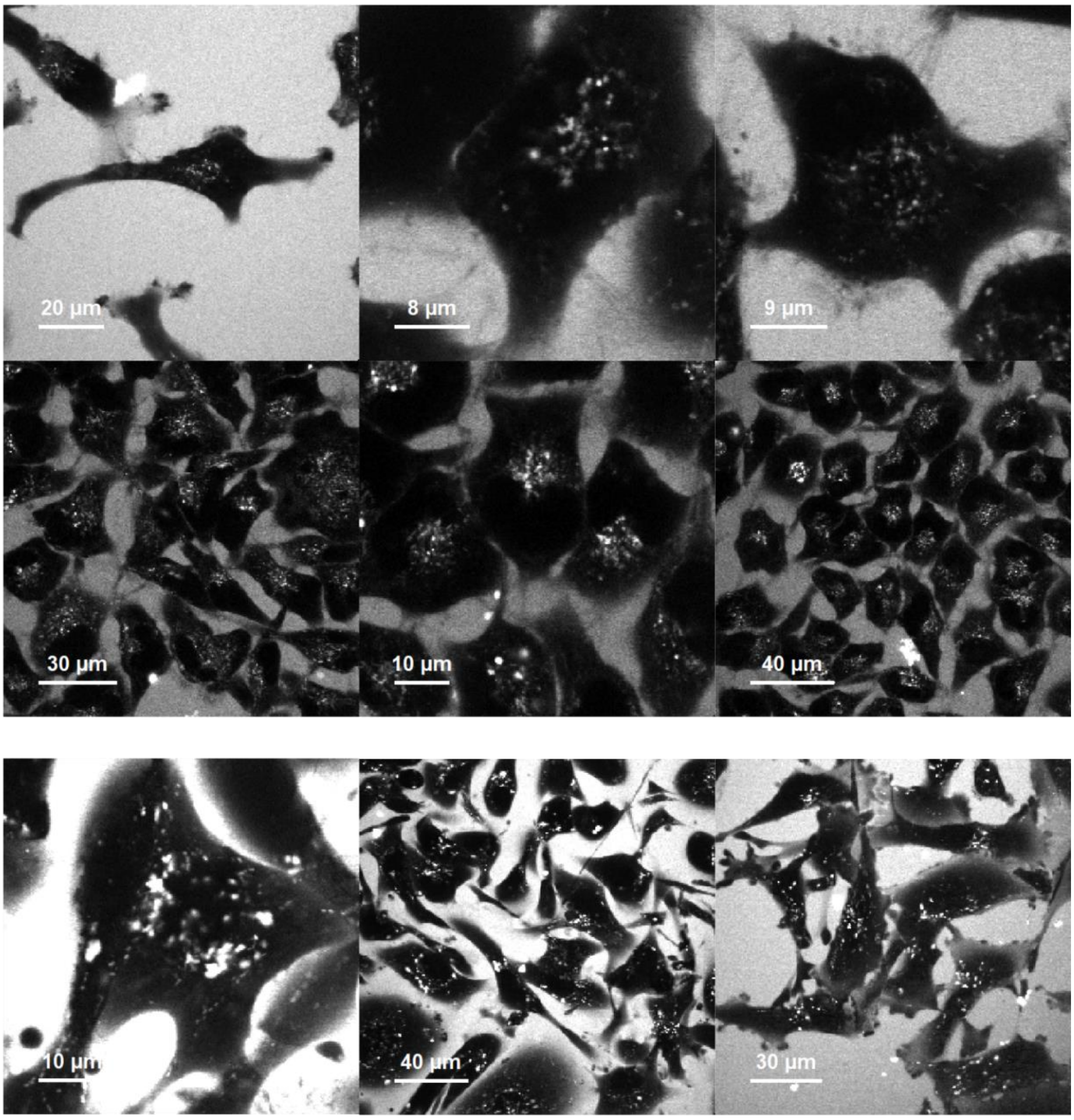

Figure S2. Confocal images of HeLa and MDA-MB-231 cells incubated with the labeled bevacizumab. In the case of the HeLa cell line, we also included the pictures taken after 48h with the 100 nM of the tested drug.

SI3. Detailed FLIM analysis

We used FLIM as one of the methods to examine the internalization of bevacizumab into living cells. Due to the low number of the uptaken molecules, autofluorescence of the cells could interfere with the signal. FLIM analysis allowed us to distinguish the autofluorescence signal from the signal emitted by the tested drug.

Exemplary, detailed FLIM analysis with the representative fluorescence intensity decay curve, the instrument response function (IRF), the double-exponential tailfit function used as a fit model, and residuals for bevacizumab in a buffer are presented in Figure S3A. Values of all fitted parameters are summarized in Table S1.

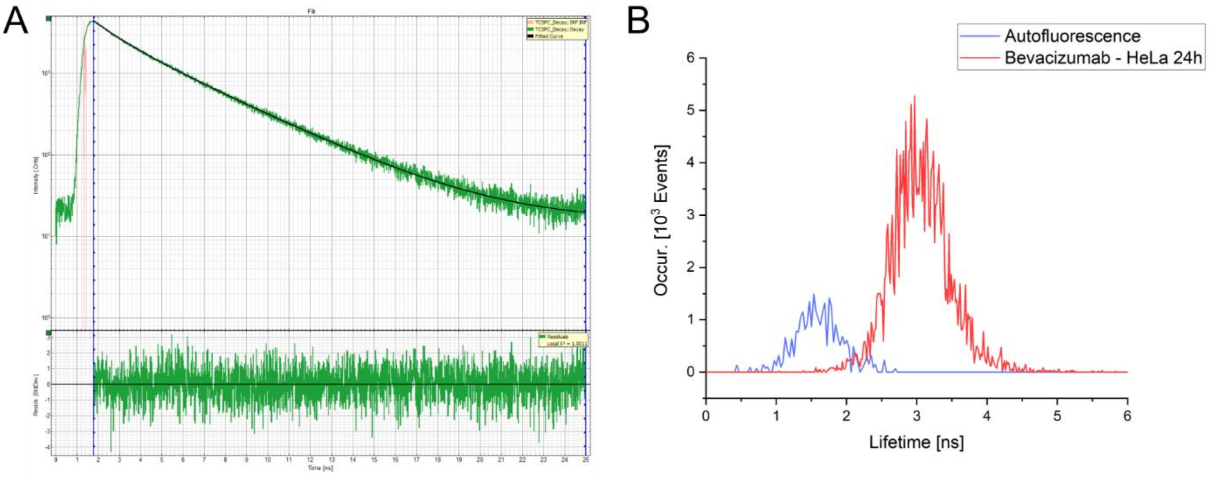

Figure S3. Detailed FLIM analysis. A) Exemplary time-correlated photon counting (TCPC) decay curve for fluorescent bevacizumab in a buffer with instrument response function (IRF) indicated. The bottom panel represents residuals. B) Histogram of fluorescence lifetime for HeLa cells' autofluorescence (blue) and bevacizumab-Atto 488 inside HeLa cells after 24h of incubation.

We also compared histograms of fluorescence lifetimes for cells' autofluorescence with those for internalized bevacizumab (Figure S3B). We clearly showed that the fluorescence lifetime for autofluorescence is around 1.5 ns, while for bevacizumab – about 3 ns. In this way, we excluded the possibility of losing photons from Atto 488 with fluorescence lifetimes shorter than 2.6 ns.

Table S1. Values of fitted with 2-exponential tailfit parameters for bevacizumab in a buffer. A_1 corresponds to the fraction of the first lifetime, A_2 – the fraction of the second lifetime, τ_1 stands for the fluorescence lifetime of the first component, τ_2 is the fluorescence lifetime of the second component, and $Bkgr_{Dec}$ corresponds to the background decay.

Parameter	2-exponential tailfit
A_1 [kCnts]	3.09 ± 0.03
A_2 [kCnts]	1.33 ± 0.03
τ_1 [ns]	3.53 ± 0.02
τ_2 [ns]	1.24 ± 0.03
$Bkgr_{Dec}$ [Cnts]	16.00 ± 0.35

SI4. The size of fluorescent bevacizumab. No interaction between bevacizumab and FBS components

We performed FCS measurements in a buffer to determine the size of fluorescently labeled bevacizumab. The results obtained are shown in Table S2. The hydrodynamic radius of the fluorescent drug was 6.16 ± 0.18 nm. In previous studies, the hydrodynamic radius of the bevacizumab was measured to be in the range of 4.58 nm to 6.5 nm.[4–8] Our results for the fluorescent drug fall within this range. The size of the drug molecule also directly determines its mechanism of entry into the cell as far as passive diffusion is determined by the size of the channels located on the cell membrane.[9] Bevacizumab, with a diameter of 12 nm, does not passively enter through cell membrane channels. Therefore, the only way for the drug to enter the cell is active uptake (understood as active endocytosis) or constitutive endocytosis (a type of endocytosis that occurs continuously).[10]

In the context of literature reports on the impact of the FBS presence in the culture medium on the internalization of bevacizumab into cells, we examined whether the tested drug could form complexes with FBS components. We measured the diffusion coefficients of bevacizumab and bevacizumab-VEGF mixture and could not find significant changes in the measured values ($p>0.05$, t-test, $N>15$). We demonstrated that the hydrodynamic radius of bevacizumab incubated with 10% FBS does not change its size (Table S2). Thus, we excluded the drug interactions with FBS components.

Table S2. Diffusion coefficients and hydrodynamic radii of bevacizumab alone, bevacizumab incubated with 10% FBS, and the complex between the tested drug and the VEGF-A (measurements performed in a buffer and cell culture media). The table also included the brightness method results. The results came from three independent repeats, and the errors indicate standard deviations. Statistical analysis (t-test, $N>15$) proved that there is a significant change in diffusion coefficients between bevacizumab in the buffer and the culture media ($p<0.0005$). However, no significant differences were got during culture time or between cell lines.

**Parameter (36˚C)**	**Bevacizumab**	**Bevacizumab incubated 1h with 10% FBS**	**Bevacizumab incubated for 1h with VEGF-A (in a buffer)**	**Bevacizumab incubated for 24h with VEGF-A (in a buffer)**	**HeLa culture medium**			**MDA-MB-231 culture medium**		
					**1h incubation**	**24h incubation**	**48h incubation**	**1h incubation**	**24h incubation**	**48h incubation**
Diffusion coefficient [$\mu m^2/s$]	49.00 ± 1.41	49.13 ± 0.91	50.82 ± 1.04	50.40 ± 1.40	61.01 ± 2.62	59.66 ± 3.32	56.57 ± 2.41	54.97 ± 4.26	58.99 ± 4.16	57.00 ± 4.49
Hydrodynamic radius [nm]	6.16 ± 0.18	6.15 ± 0.11	5.94 ± 0.12	5.99 ± 0.17	5.08 ± 0.22	5.20 ± 0.29	5.48 ± 0.23	5.48 ± 0.23	5.26 ± 0.37	5.44 ± 0.43
Brightness [cnts/s]	29 734		27 548							
Partition coefficient [-]						$(1.32 \pm 0.72) \cdot 10^{-5}$			$(2.25 \pm 1.27) \cdot 10^{-5}$	$(2.48 \pm 1.08) \cdot 10^{-5}$

SI5. Quantitative data of FCS measurements performed inside HeLa and MDA-MB-231 cells

After fitting FCS curves obtained directly inside both tested cell types, we received quantitative information on the movement of the vesicles and the drug closed inside these vesicles. A diffusion coefficient describes the movement. In this way, we proved that the FCS technique could be successfully used in monitoring vesicles' active transport. Values of four different parameters with the division on the cell type are presented in the table below.

Table S3. The values of parameters obtained from fitting the FCS data using Equation S5. The measurements were performed inside HeLa and MDA-MB-231 cells. D_M represents the diffusion coefficient of the vesicle, a - the radius of the vesicle, D_m corresponds to the diffusion coefficient of the drug closed inside the vesicle, and v is the velocity forcing vesicle. The errors are standard deviations.

**Parameter**	**HeLa cells after 24h incubation with the drug**	**MDA-MB-231 cells after 24h incubation with the drug**	**MDA-MB-231 cells after 48h incubation with the drug**
**D_M [$\mu m^2/s$]**	0.03 ± 0.10	0.38 ± 0.33	0.03 ± 0.01
**a [μm]**	0.24 ± 0.06	0.25 ± 0.07	0.28 ± 0.09
**D_m [$\mu m^2/s$]**	7.89 ± 3.22	10.43 ± 9.20	6.31 ± 1.2
**v [μm/s]**	1.33 ± 1.64	2.53 ± 2.80	0.81 ± 0.76

SI6. Osmotic shock-enhanced pinocytosis

Pinocytosis is an example of active transport dedicated to small molecules' transport. Pinocytosis, as well as endocytosis, can be constitutive, which means that it occurs continuously. One of the methods used to enhance pinocytosis is the application of osmotic shock. In this process, the cell is first placed in the hypertonic solution (with high osmotic pressure) and then moved to the hypotonic one (with low osmotic pressure). The whole procedure takes only 15 minutes, and during that time, cells take up a compound present in the hypertonic medium. The mechanism based on the osmotic shock is widely used for the intracellular delivery of different kinds of probes.[11] Here we showed that MDA-MB-231 cells, after osmotic shock-enhanced pinocytosis, are fully filled the vesicles (Figure S4). Comparing the confocal images presented in Figure S4 with the pictures shown in Figure 2 or S2, there is no doubt that the fluorescent bevacizumab is not transported *via* active uptake, understood as endocytosis/pinocytosis. The number of vesicles inside tested cells incubated 24 or 48h with the drug is incomparably smaller than the number of vesicles after osmotic shock-enhanced pinocytosis.

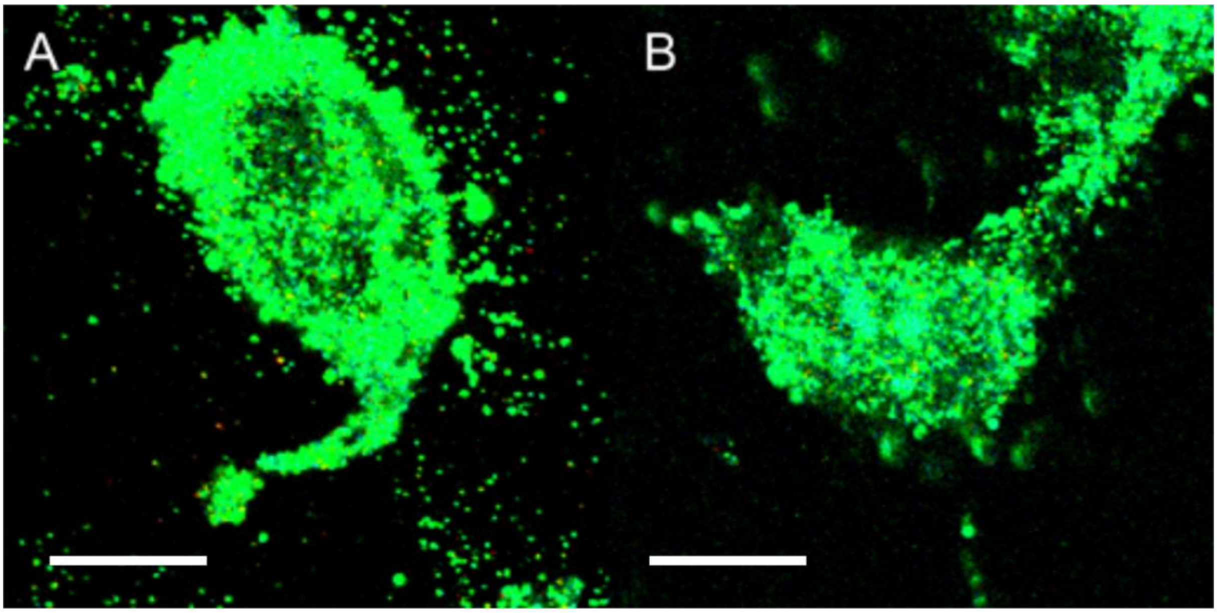

Figure S4. Confocal images of MDA-MB-231 cells filled with vesicles after osmotic shock-mediated intracellular delivery. The images were taken after 30 minutes after the osmotic shock procedure. The hypertonic solution contained 1.2 M of sucrose. A) Intracellular delivery of TRITC-labeled dextran 20 kDa [11]. B) Intracellular delivery of TRITC-labeled dextran 155 kDa. Scale bar = 10 µm.

SI7. Active transferrin uptake

We conducted a control experiment, incubating HeLa cells with transferrin at 50 nM and 200 nM for about 1h. Confocal images are shown in Figure S5. Transferrin is a protein that regulates iron transport and is a model example of a molecule transported by clathrin-dependent endocytosis.[12] As seen in Figure S5, after one hour of incubation with transferrin, vesicles corresponding to endosomes were visible inside the HeLa cells. Their number (regardless of protein concentration) was significantly higher than the number of vesicles visible in HeLa cells after 24h incubation with 500 nM of fluorescent bevacizumab. By performing such an experiment, we additionally proved that the tested antibody could not be transported by active uptake. If this were the active uptake, after just 1h of incubation, endosomes would be visible in the cells. In contrast, endosomes are present inside the cell only after 24h (Figure S2). This time scale provides additional confirmation that bevacizumab was just taken up by the cells as a result of constitutive endocytosis occurring all the time.

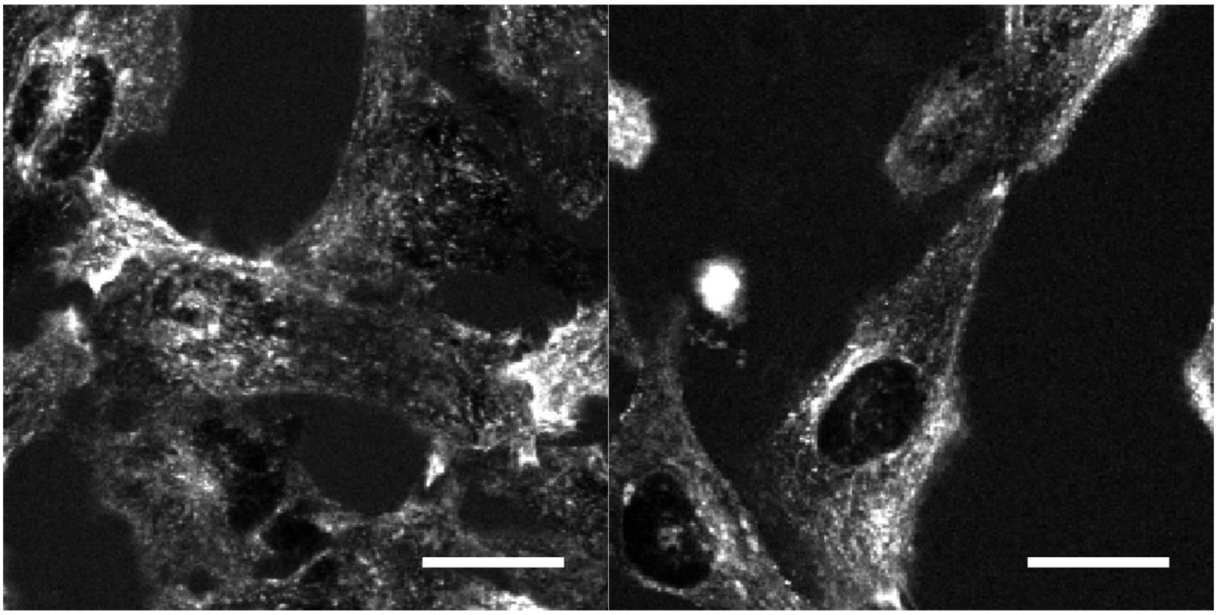

Figure S5. Confocal images of HeLa cells incubated for 1h with transferrin at 200 nM (left image) and 50 nM (right image). Scale bar = 10 µm.

SI8. Exemplary time traces and corresponding autocorrelation curves. The photobleaching analysis

The FCS autocorrelation curve is the result of mathematical processing of the fluorescence fluctuations over time. The course of fluorescence over time (time trace) determines the shape of the FCS curve. Below we provide exemplary time traces and corresponding autocorrelation curves for HeLa cells after 24h incubation and MDA-MB-231 cells after 24h and 48h incubation. Example plots are included for both variants: when the detection position was the cytosol (no signal from the drug) and when the confocal focus was located within the presence of vesicles.

We also excluded the photobleaching phenomenon using the exemplary time traces given in Figure S6. The occurrence of photobleaching, a common problem in FCS measurements, impacts the shape of the FCS curve - the additional characteristic decay time or the lack of convergence of the FCS curve to zero may appear. We clearly excluded photobleaching based on comparing the average fluorescence intensity in the first and last seconds of the measurement. Thus, we have proved that the obtained FCS curves reflect the movement of the vesicle and the enclosed fluorophore.

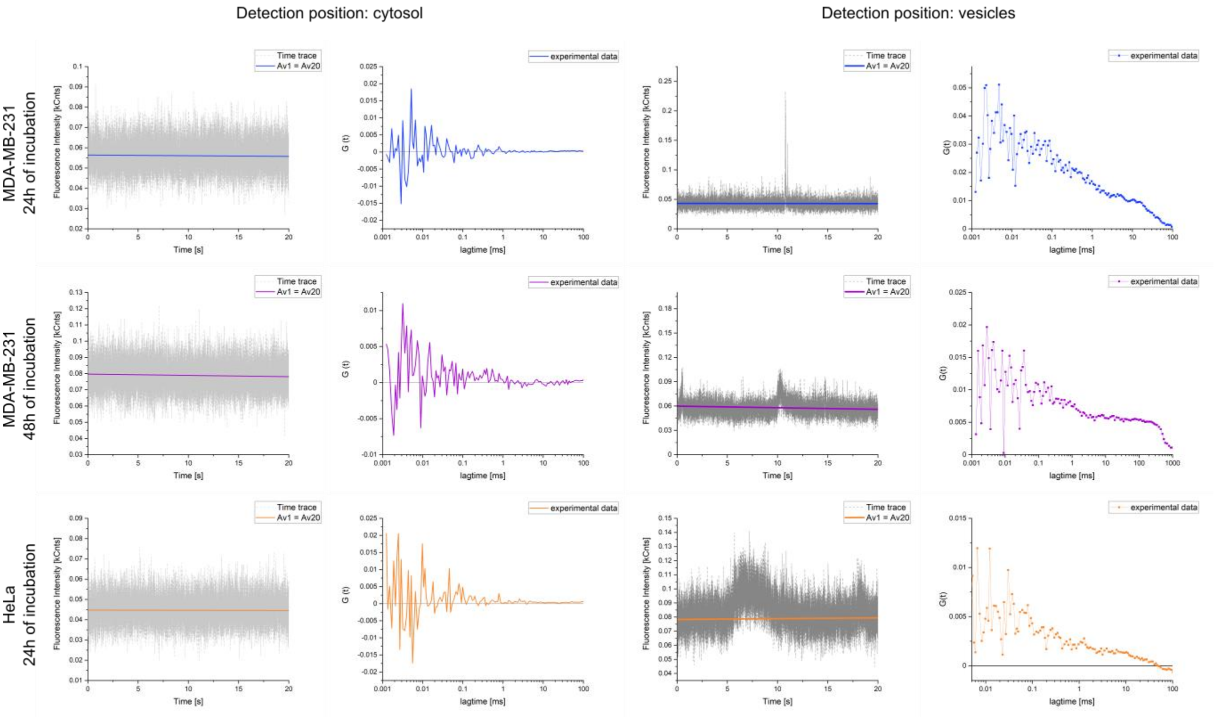

Figure S6. Exemplary recordings of fluorescence intensity over time (time traces) and corresponding FCS autocorrelation curves both in the cytosol and within the vesicle occurrence. Results presented for HeLa after 24h incubation with the drug and for MDA-MB-231 after 24h and 48h incubation.

SI9. The possible impact of ECM proteases and nanoviscosity changes on the bevacizumab size

We noted bevacizumab size decrease in the culture medium surrounding living cells (extracellular measurements) compared to measurements in a buffer. We speculate that a smaller hydrodynamic radius in the culture medium can be associated with the existence of the extracellular matrix (ECM). ECM is full of proteases, which can decompose the tested drug. However, it seems that the effect of digestive enzymes should give a larger change in drug size than observed. Another explanation for the drug size decrease during extracellular measurements could be a local decrease in ECM viscosity for 2D cell cultures. Hydrodynamic radius is a parameter that strongly depends on the viscosity of the medium. Previous studies showed the ECM nanoviscosity in 3D cell cultures depending on the length scale.[13] For a probe of 6 nm radius, the experienced viscosity is about 1.5 times higher than the water viscosity, so the hydrodynamic radius of such cargo (Equation S4) would be much smaller than the obtained one (Table S2). However, still, these speculations concern the 3D cell cultures, not the 2D cells measured in this paper.

SI10. The fluorescence intensity of bevacizumab and its complex with VEGF

The target of bevacizumab is to bind to the VEGF factor. We checked whether the bevacizumab, after binding to VEGF, changes its size enough to make these changes visible during FCS experiments. Measurements in a PBS solution containing the drug and the VEGF-A at a stoichiometric ratio of 1:2 showed no significant differences in diffusion coefficient between the drug alone and the drug incubated with the VEGF-A for 1h and 24h at 36˚C.

We performed analogous measurements in the culture medium surrounding living cells, both HeLa and MDA-MB-231, to ensure that the results would be the same as in a buffer. All results are summarized in Table S2. Diffusion coefficients measured in the cell culture medium are statistically significantly higher ($p<0.0005$) than those measured in buffer solution. The differences may be due to structural changes of the drug caused by the extracellular matrix. Extracellular matrix (ECM) is a

network composed of collagen fibers, enzymes, proteoglycans, and many other macromolecules, which play an essential role in cell attachment, cell-cell communication, or cell growth. The ECM is a dynamic structure undergoing constant changes.[14] At the same time, it is known that the protein structure (including an antibody) can change depending on electrostatic, hydrophobic forces, or hydrogen bonds. Even weak interactions with biomolecules present in the ECM can cause slight conformation changes compared with the situation occurring in the buffer. This issue raises the question of how properly select buffers for biochemical studies to most accurately reflect living cells' conditions. The possible effect of proteases and nanoviscosity changes was also excluded (please see section SI9).

On the other hand, the hydrodynamic radius also depends on the diffusion coefficient (Equation S4), which in turn depends on the size of the confocal focus. However, it can certainly be excluded that the observed decrease in the size of the tested drug is due to a change in the confocal focus size. Before each measurement, the focus size is determined during calibration, and the calculated diffusion coefficients (Equation S3) and subsequent hydrodynamic radii (Equation S4) take the obtained focus size into account. Considering the abovementioned issues, the question of why the bevacizumab size is smaller when the tested drug is present in the ECM remains open.

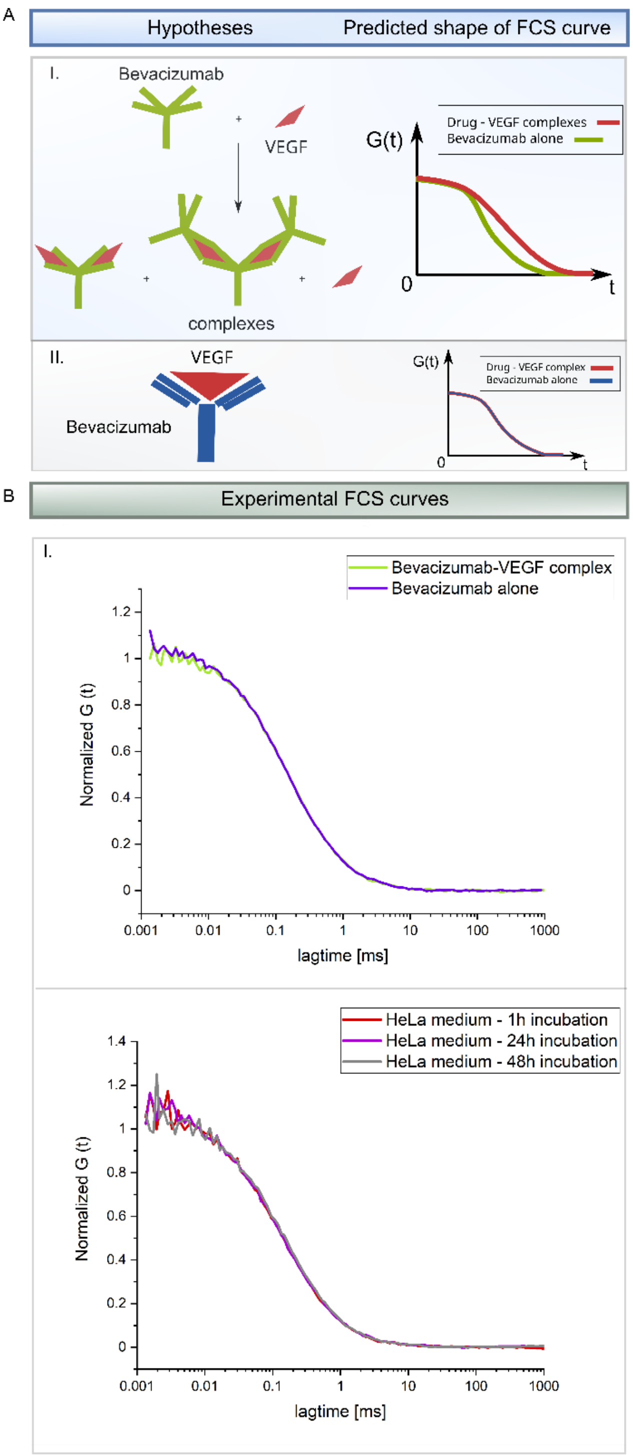

Figure S7. Theoretical possibilities of bevacizumab binding to VEGF vs. experimentally obtained FCS curves showing no change after the drug binds to VEGF. A) Different theories of what bevacizumab complex with VEGF looks like. I) The mechanism of VEGF binding to bevacizumab proposed by Chang and others.[15] One drug molecule binds two molecules of The VEGF. The concatenation (formation of supramolecular complexes) is also possible. II) Simple scheme presenting the bevacizumab complex with the VEGF deposited in the PDB database.[16] B) The shape of experimental FCS autocorrelation curves as an indicator of probe size changes: I) the shape of FCS curves obtained from measurements in a buffer for bevacizumab alone and its complex with the VEGF. No changes were visible. II) the shape of FCS curves representing the results obtained from the HeLa culture medium after different incubation times with bevacizumab. As in a buffer, we did not see any changes in FCS curve shapes, indicating no differences in bevacizumab size after binding the VEGF.

Our FCS results show that the size of the bevacizumab-VEGF complex and the drug alone is the same (the changes are within the errors). The shape of the FCS curves (Figure S7B) confirmed the

calculations. In the literature, there are two hypotheses regarding the complex structure between VEGF-A and bevacizumab. Chang and others described the first one:[15] the authors claim that in a bevacizumab sample with an excess of VEGF, one drug molecule will bind two molecules of the factor. The proposed process of the complex formation is shown in Figure S7A (I). Moreover, the formation of supramolecular complexes is possible. What is also essential, Chang and others[15] obtained experimental data (a shift in peaks on the chromatogram), indicating an increase in size when the VEGF factor was added to the sample. Our FCS results excluded the possibility of the formation of larger complexes as we did not observe any additional component with a radius bigger than the tested drug. The presence of such big complexes would be reflected in the FCS curves' shape (Figure S7A). Consequently, this would determine using a two-component diffusion model to fit the experimental data.

An alternative way of VEGF binding can be deduced from a 3D structure of the bevacizumab Fab mutant in a complex with VEGF in the PDB database.[16] The structure shows that the VEGF factor is located between two Fab fragments of the antibody. In this case, the stoichiometric ratio is one molecule of the VEGF per 1 molecule of the drug (Figure S7A, II).

Considering the molecular masses of VEGF-A and drug (149 kDa and 45 kDa, respectively) and the reports proving that VEGF incorporates between specific antibody fragments, it can be assumed that the results obtained from FCS measurements indicate the scenario that bevacizumab does not change its size after VEGF binding.

The FCS technique could not confirm VEGF binding to bevacizumab due to the lack of apparent size changes between the complex and the drug itself. Therefore, we used our previously described brightness method[3,17,18] to track the binding between the drug and its target. The brightness method is based on detecting single-photon changes in fluorescence intensity. The conceptual scheme explaining the principle of the brightness method is shown in Figure S8.

Upon binding of VEGF, the brightness of the drug molecule changes. The brightness of the bevacizumab molecule alone is higher than that of the drug-VEGF complex. The obtained experimental results are shown in Table S2 (the brightness of bevacizumab alone = 29 734; the brightness of bevacizumab-VEGF complex = 27 548). At a stoichiometric ratio of 1:30 (bevacizumab : VEGF), we observed a 7% decrease in the fluorescence intensity of the drug after binding to VEGF. It is worth mentioning that the percentage changes (7% in our case) in fluorescence intensity after binding with the VEGF strongly depend on the localization of dye molecules in the drug after the labeling procedure. Some may be present at the binding site with VEGF (in which case the intensity change will be more clear). At the same time, some may be localized elsewhere in the antibody, translating into a lower brightness decrease. Nevertheless, we proved that the fluorescent bevacizumab binds to the VEGF in a buffer. We could monitor this interaction using the brightness method.

SI11. Brightness method as a method for monitoring the drug-target interactions

In the paper, we used the previously described brightness method[3,17,18] to prove bindings between bevacizumab and its target – VEGF. In Figure S8, we presented a simple scheme explaining the principle of this technique. The brightness method is based on measuring the fluorescence intensity. The intensity of the probe containing only fluorescent bevacizumab, I_1, should be higher than the intensity of the complex between the tested drug and the non-fluorescent VEGF, I_2. The results obtained from the brightness method are shown in Table S2. We observed that after binding to the VEGF, the intensity of the drug decreased by 7%. The tested stoichiometric ratio was 1:30 (bevacizumab : VEGF).

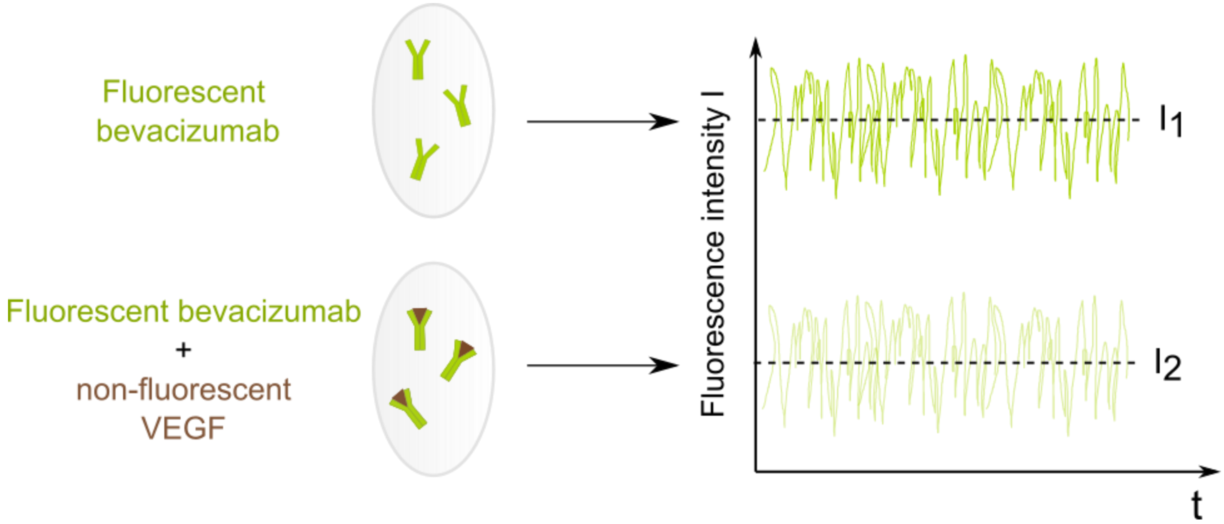

Figure S8. The scheme of the brightness method. The technique is based on measuring fluorescence intensity. If only the fluorescent drug is present in the confocal focus, its I_1 intensity is higher than the fluorescence intensity of the bevacizumab complex with unlabeled VEGF, I_2 ($I_1 > I_2$).

SI12. Calculation of bevacizumab intracellular concentration

As a part of the study, we calculated the partition coefficient to define the effectiveness of bevacizumab internalization. The partition coefficient is the drug's intracellular concentration ratio to the extracellular concentration. The extracellular concentration was determined using FCS measurements, while we calculated the intracellular concentration of bevacizumab based on FLIM/confocal images. A scheme precisely explaining the way of intracellular concentration determination is presented in Figure S9.

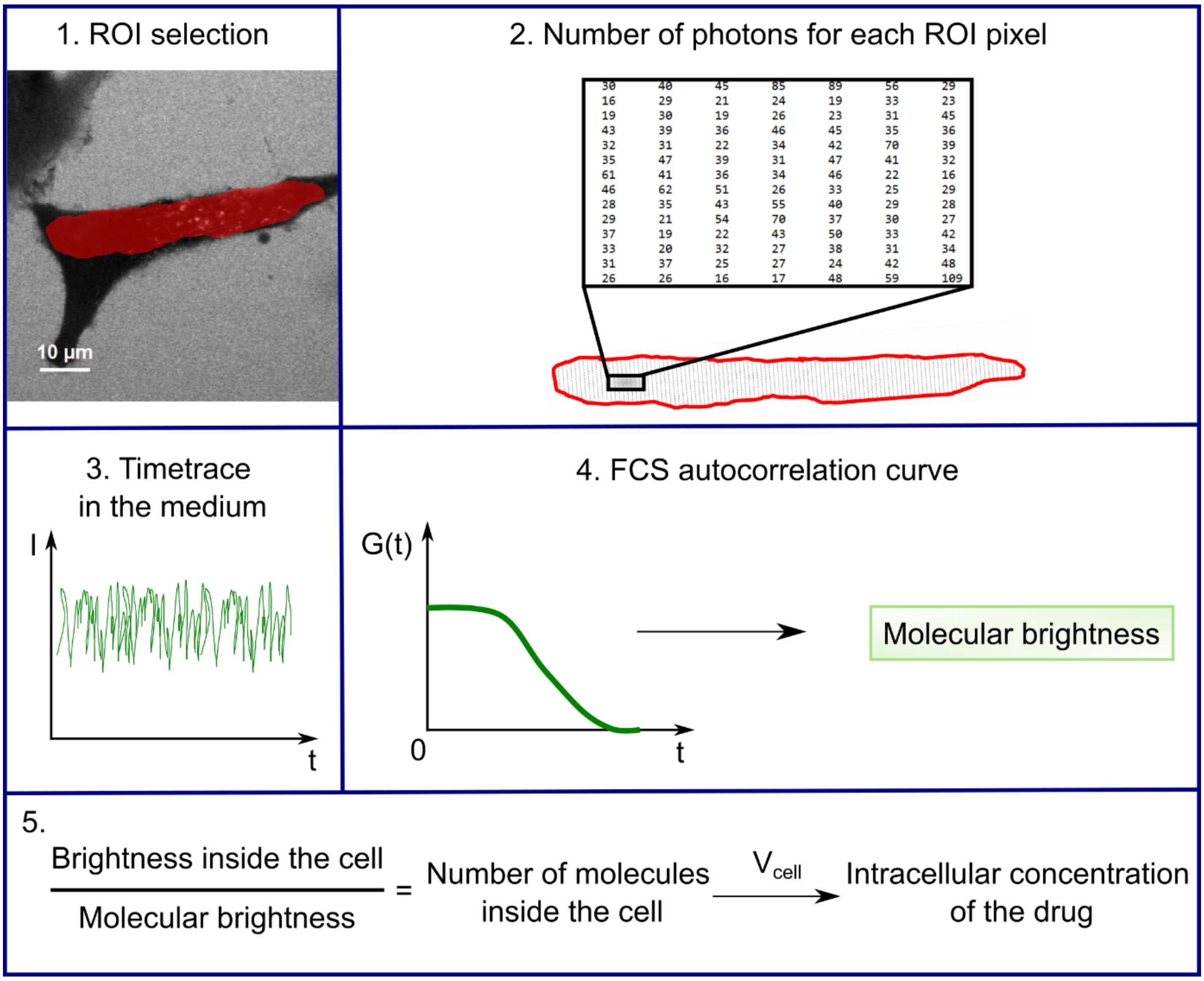

Figure S9. A scheme explaining the way of the bevacizumab intracellular concentration calculation. In the first step, we selected the ROI of each confocal/FLIM image. Then, using SymphoTime software, we obtained the number of photons for each pixel within the selected ROI. Performing reference FCS measurements in the medium, we acquired the FCS autocorrelation curves based on Timetrace (the fluorescence intensity dependence on the time). FCS curves contained information about the drug's molecular brightness (number of photons per molecule). In the last step, we calculated the number of drug molecules inside the tested cell as the ratio of the drug brightness inside the cell to the drug molecular brightness, and, considering the cell volume (set as $2 \cdot 10^3$ µm^3), we obtained the intracellular concentration of bevacizumab.

SI13. Labeled bevacizumab is toxic to HeLa cells

The morphological changes observed by imaging for the HeLa cell line incubated 48h with the labeled drug suggested the cytotoxicity of bevacizumab against this tumor type. We examined the cytotoxicity of fluorescent bevacizumab against both cell lines, MDA-MB-231 and HeLa, to quantitatively confirm the imaging results. The cytotoxicity of the labeled drug was tested using the alamarBlue® assay. As shown in Figure S10 (C, D), fluorescent bevacizumab at the highest tested concentrations (500 nM and 250 nM) killed cervical cancer cells. The IC_{50} value for HeLa cells was 149.64 nM.

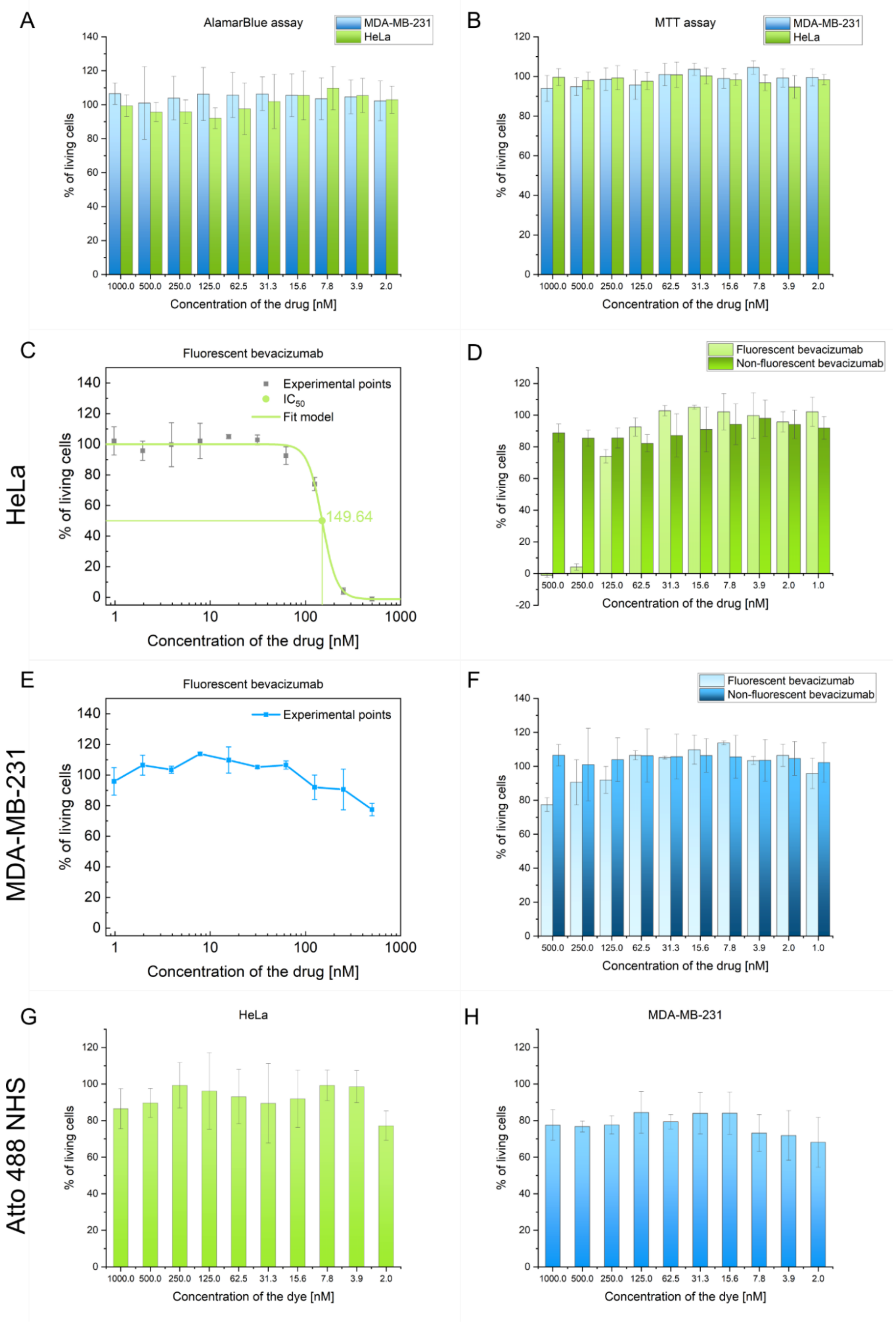

Figure S10. Cytotoxicity of fluorescent and non-fluorescent bevacizumab (including the effect of only dye) against HeLa and MDA-MB-231 cells after 48h incubation. The green color represents the results obtained for the HeLa cell line, and the blue

one - shows the data for MDA-MB-231. A) Cell viability dependence on the non-fluorescent bevacizumab concentration, determined by alamarBlue® assay. B) Cell viability dependence on the non-fluorescent bevacizumab concentration, determined by MTT assay. C) HeLa cells viability incubated with the fluorescent drug as a function of compound concentration, with IC_{50} value based on alamarBlue® assay. D) Cytotoxicity comparison of the fluorescent and non-fluorescent drug against HeLa cell line. E) MDA-MB-231 cells viability incubated with the fluorescent drug as a function of compound concentration, with IC_{50} value based on alamarBlue® assay. F) Cytotoxicity comparison of fluorescent and non-fluorescent bevacizumab against MDA-MB-231 cell line. G) Cytotoxicity of only Atto 488 NHS ester (the used dye) against HeLa cells determined using MTT assay. H) Cytotoxicity of only Atto 488 NHS ester against MDA-MB-231 cell line defined using MTT assay.

In comparison, triple-negative breast cancer cells (MDA-MB-231) incubated with 500 nM and 250 nM fluorescently labeled bevacizumab remained 77% and 90% alive. The slight decrease in MDA-MB-231 cell viability seen in Figure S10 (E, F) for the three highest drug concentrations may suggest some small cellular response to the tested compound. However, the decrease is still within the error range. Thus, the alamarBlue® assay results confirmed the FLIM observations.

We also investigated whether attaching a dye molecule to a drug molecule affects its pharmacological properties. Thus, we tested the potential cytotoxicity of dye-free bevacizumab against both tested cell lines (Figure S10: D, F). In this case, we used two different cytotoxicity assays: MTT and alamarBlue. Both applied assays showed that unlabeled bevacizumab has no cytotoxicity against any tested tumor types. For the MTT assay, the viability of both cell lines is maintained at 100%. AlamarBlue®, a bit more sensitive than the MTT assay, showed a slight decrease in cell viability of the HeLa line for the highest bevacizumab concentrations (Figure S10: A, B). Still, this decrease was at the 20% level and was not experimentally meaningful.

Our results concerning triple-negative breast cancer are consistent with the literature data. Emlet and others reported a slight decrease in the MDA-MB-231 cell line viability for unlabeled bevacizumab at concentrations up to 670 nM.[19] This lack of response in triple-negative breast cancer cells' viability to non-fluorescent bevacizumab may be due to a significant decrease in the expression level of the drug target - VEGF factor, after 24h incubation of MDA-MB-231 cells with the drug.[20]

Moreover, the obtained cytotoxicity results clearly show that labeling bevacizumab alters its clinical potential. These observations are probably strongly connected with a different equilibrium constant between the drug and its action target - the VEGF-A factor. The labeled drug-VEGF-A complex's equilibrium constant is likely higher than the one for unlabeled bevacizumab. In turn, the value of the equilibrium constant directly translates into therapeutic relevance and pharmacological properties. We also checked the cytotoxicity of the dye itself (Atto 488 NHS ester) to be sure that the noted toxicity of the labeled drug was not due to the toxicity of the dye itself. The dye was not harmful to any tested cells, even at the highest tested concentration equal to 1 μM. The viability of HeLa cells exposed to Atto 488 NHS was at 90% for all tested concentrations (Figure S10G). In the case of MDA-MB-231 cells, about 80% of cells treated with Atto 488 NHS remained alive independent of the dye concentration (Figure S10H).

SI14. The cell height in 2D culture

In FLIM measurements, we performed one-layer scanning. The confocal focus was positioned at the height of the presence of vesicles - the only sites of bevacizumab. These vesicles were mainly located around the cell nucleus, whose position determines the cell's height. Changing the focus position in the 'Z' dimension resulted in a loss of their visibility.

We conducted AFM analysis for HeLa cells in 2D culture to estimate the exact height of the HeLa cell. The results are presented in Figure S11.

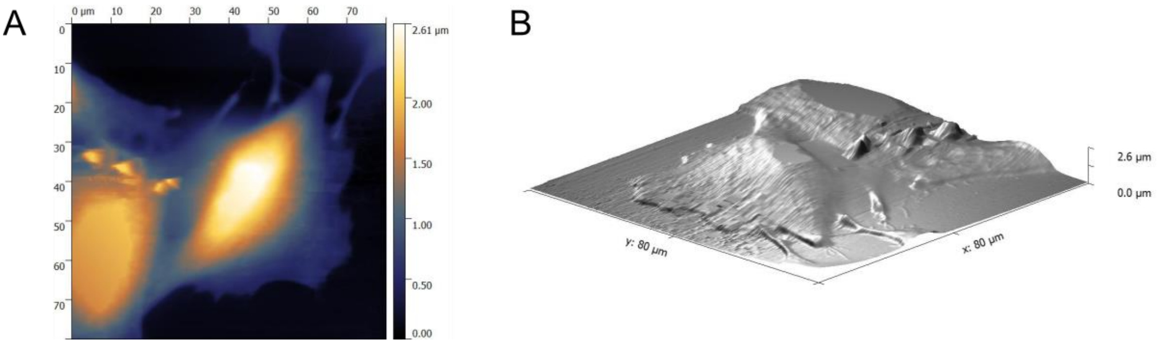

Figure S11. AFM analysis for HeLa cells under 2D conditions, flattened on a glass surface. A) An AFM color map of HeLa cells. B) 3D AFM image of HeLa cells.

AFM analysis showed that the HeLa cell height in 2D culture, flattened on a glass surface, equals 2.6 µm. The typical height of the ellipsoidal confocal focus in FCS/FLIM analysis is around 2.3 µm. Thus, the height of the confocal focus, defined during calibration, is ~ 90% of the height of the entire cell.

SI15. FLIM images of HeLa cells incubated with 100 nM of bevacizumab

In all experiments presented in the manuscript, the extracellular bevacizumab concentration was 500 nM. Thus, in the confocal focus, placed in the medium, there were several tens (~50) of fluorophores. Assuming that the concentration of fluorophores in the vesicle is the same as in the medium, there are about 20 fluorescent molecules in a vesicle of 0.25 µm radius. Thus, the signal from the vesicle centered in the confocal volume would be ~2.5 times weaker than from the medium.

To confirm that after 1h incubation, vesicles were not present inside cells regardless of the drug concentration in the medium, we performed imaging for HeLa cells incubated 1h with 100 nM bevacizumab (5-fold lower concentration than in the experiments described in the manuscript). As a control, we performed imaging for autofluorescence of HeLa cells, with the same laser power. The images presented in Figure S12 clearly show that the reddish signal recorded inside the HeLa cells after 1h incubation with the drug corresponds to the autofluorescence signal.

In addition, we also show that bright vesicles were visible in HeLa cells even at a concentration of the drug in the medium equal to 100 nM (Figure S12C). Moreover, after 48h of incubation, the HeLa cells remained alive (based on the morphology), which is consistent with the results obtained from cytotoxicity assays (Supporting Information, SI13).

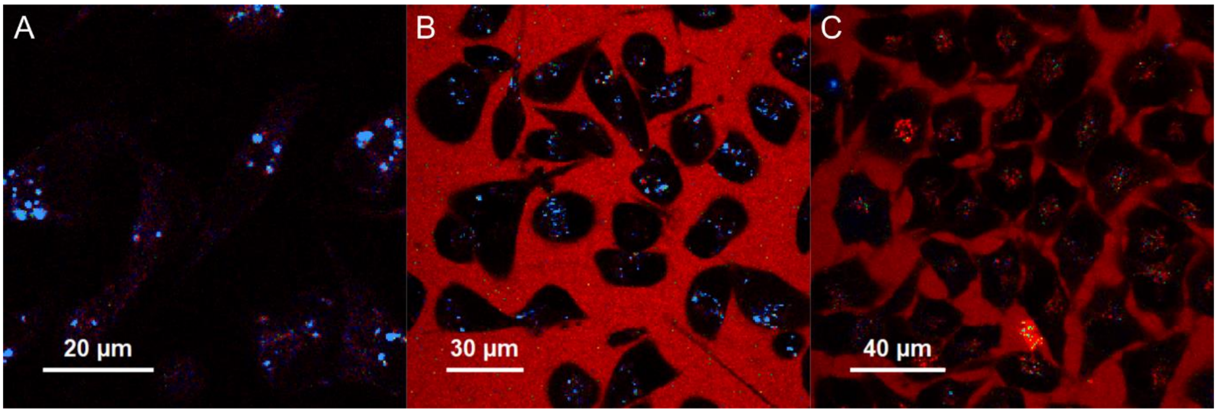

Figure S12. HeLa cells incubated with a 100 nM concentration of bevacizumab. A) HeLa cells autofluorescence (laser power = 10 µW). B) HeLa cells incubated for 1h with 100 nM of bevacizumab. The reddish signal in the cells corresponds to the autofluorescence signal. C) HeLa cells incubated for 48h with 100 nM of fluorescent bevacizumab. Drug-filled vesicles located around the nucleus are visible.

References

(1) Kalwarczyk, T.; Kwapiszewska, K.; Szczepanski, K.; Sozanski, K.; Szymanski, J.; Michalska, B.; Patalas-Krawczyk, P.; Duszynski, J.; Holyst, R. Apparent Anomalous Diffusion in the Cytoplasm of Human Cells: The Effect of Probes' Polydispersity. *J. Phys. Chem. B* **2017**, *121* (42), 9831–9837.

(2) Ochab-Marcinek, A.; Hołyst, R. Scale-Dependent Diffusion of Spheres in Solutions of Flexible and Rigid Polymers: Mean Square Displacement and Autocorrelation Function for FCS and DLS Measurements. *Soft Matter* **2011**, *7* (16), 7366–7374.

(3) Karpińska, A.; Pilz, M.; Buczkowska, J.; Żuk, P. J.; Kucharska, K.; Magiera, G.; Kwapiszewska, K.; Hołyst, R. Quantitative Analysis of Biochemical Processes in Living Cells at a Single-Molecule Level: A Case of Olaparib-PARP1 (DNA Repair Protein) Interactions. *Analyst* **2021**, *146* (23), 7131–7143.

(4) Li, S. K.; Liddell, M. R.; Wen, H. Effective Electrophoretic Mobilities and Charges of Anti-VEGF Proteins Determined by Capillary Zone Electrophoresis. *J. Pharm. Biomed. Anal.* **2011**, *55* (3), 603–607.

(5) Paul, M.; Vieillard, V.; Roumi, E.; Cauvin, A.; Despiau, M. C.; Laurent, M.; Astier, A. Long-Term Stability of Bevacizumab Repackaged in 1mL Polypropylene Syringes for Intravitreal Administration. *Ann. Pharm. Fr.* **2012**, *70* (3), 139–154.

(6) Wen, H.; Hao, J.; Li, S. K. Characterization of Human Sclera Barrier Properties for Transscleral Delivery of Bevacizumab and Ranibizumab. *J. Pharm. Sci.* **2013**, *102* (3), 892–903.

(7) Khalili, H.; Sharma, G.; Froome, A.; Khaw, P. T.; Brocchini, S. Storage Stability of Bevacizumab in Polycarbonate and Polypropylene Syringes. *Eye* **2015**, *29* (6), 820–827.

(8) Hirvonen, L. M.; Fruhwirth, G. O.; Srikantha, N.; Barber, M. J.; Neffendorf, J. E.; Suhling, K.; Jackson, T. L. Hydrodynamic Radii of Ranibizumab, Aflibercept and Bevacizumab Measured by Time-Resolved Phosphorescence Anisotropy. *Pharm. Res.* **2016**, *33* (8), 2025–2032.

(9) Zhang, X.; Fu, W.; Palivan, C. G.; Meier, W. Natural Channel Protein Inserts and Functions in a Completely Artificial, Solid-Supported Bilayer Membrane. *Sci. Rep.* **2013**, *3* (2196), 1–7.

(10) Rennick, J. J.; Johnston, A. P. R.; Parton, R. G. Key Principles and Methods for Studying the Endocytosis of Biological and Nanoparticle Therapeutics. *Nat. Nanotechnol.* **2021**, *16* (3), 266–276.

(11) Karpinska, A.; Zgorzelska, A.; Kwapiszewska, K.; Hołyst, R. Entanglement of Polymer Chains in Hypertonic Medium Enhances the Delivery of DNA and Other Biomacromolecules into Cells. *J. Colloid Interface Sci.* **2022**, *627*, 270–282.

(12) Mayle, K. M.; Le, A. M.; Kamei, D. T. The Intracellular Trafficking Pathway of Transferrin. *Biochim. Biophys. Acta* **2012**, *1820* (3), 264–281.

(13) Pilz, M.; Kwapiszewska, K.; Kalwarczyk, T.; Bubak, G.; Nowis, D.; Hołyst, R. Transport of Nanoprobes in Multicellular Spheroids. *Nanoscale* **2020**, *12* (38), 19880–19887.

(14) Lu, P.; Takai, K.; Weaver, V. M.; Werb, Z. Extracellular Matrix Degradation and Remodeling in Development and Disease. *Cold Spring Harb. Perspect. Biol.* **2011**, *3* (12), 1–24.

(15) Chang, D. P.; Burra, S.; Day, E. S.; Chan, J.; Comps-Agrar, L.; Nivaggioli, T.; Rajagopal, K. Long-Term Stability of Anti-Vascular Endothelial Growth Factor (a-VEGF) Biologics Under Physiologically Relevant Conditions and Its Impact on the Development of Long-Acting Delivery Systems. *J. Pharm. Sci.* **2020**, *110* (2), 860–870.

(16) RCSB PDB Protein Data Bank (accessed May 17, 2022).

(17) Zhou, Y.; Bielec, K.; Pasitsuparoad, P.; Hołyst, R. Single-Molecule Brightness Analysis for the Determination of Anticancer Drug Interactions with DNA. *Analyst* **2020**, *145*, 6600–6606.

(18) Bielec, K.; Bubak, G.; Kalwarczyk, T.; Holyst, R. Analysis of Brightness of a Single Fluorophore for Quantitative Characterization of Biochemical Reactions. *J. Phys. Chem. B* **2020**, *124* (10), 1941–1948.

(19) Emlet, D. R.; Brown, K. A.; Kociban, D. L.; Pollice, A. A.; Smith, C. A.; Ong, B. B. L.; Shackney, S. E. Response to Trastuzumab, Erlotinib, and Bevacizumab, Alone and in Combination, Is Correlated with the Level of Human Epidermal Growth Factor Receptor-2 Expression in Human Breast Cancer Cell Lines. *Mol. Cancer Ther.* **2007**, *6* (10), 2664–2674.

(20) EL-Hajjar, L.; Jalaleddine, N.; Shaito, A.; Zibara, K.; Kazan, J. M.; El-Saghir, J.; El-Sabban, M. Bevacizumab Induces Inflammation in MDA-MB-231 Breast Cancer Cell Line and in a Mouse Model. *Cell. Signal.* **2019**, *53*, 400–412.
